# Road transportation is associated with decreased intestinal motility in horses

**DOI:** 10.3389/fvets.2025.1647236

**Published:** 2025-08-18

**Authors:** Sharanne L. Raidal, Francesca Freccero, Ann Carstens, Sarah Weaver, Barbara Padalino

**Affiliations:** ^1^Melbourne Veterinary School, The University of Melbourne, Werribee, VIC, Australia; ^2^School of Agricultural, Environmental and Veterinary Sciences, Faculty of Science, Charles Sturt University, Wagga Wagga, NSW, Australia; ^3^Department of Veterinary Medical Sciences, University of Bologna, Bologna, Italy; ^4^School of Animal and Veterinary Sciences, The University of Adelaide, Roseworthy, SA, Australia; ^5^Faculty of Science and Engineering, Southern Cross University, Lismore, NSW, Australia; ^6^Department of Agricultural and Food Sciences, University of Bologna, Bologna, Italy

**Keywords:** transport, equine, gastrointestinal physiology, health, welfare, cortisol, stress, ultrasonography

## Abstract

**Background:**

Transportation of horses has been associated with colic and changes to the gastrointestinal microbiome. Percutaneous abdominal ultrasonography using wireless, point-of-care transducers can be used to assess gastrointestinal motility in field settings.

**Objectives:**

Characterization of intestinal motility and salivary cortisol responses of horses completing commercial transportation of 10-12h.

**Study design:**

Prospective observational study of 30 horses with diverse signalment and transport histories. Clinical parameters, sonographic assessment of intestinal motility and saliva samples, were collected before departure (Tpre or T0), after off-loading (T1) and 2h after arrival (T2).

**Results:**

After transportation, intestinal motility grades were reduced [Tpre median (IQR) composite motility grade: 8 (7-10), T1: 6 (4-7), T2: 7 (6-7); *p* < 0.001] and qualitative changes were observed in small intestinal sonographic appearance. High ambient temperatures during transport were associated with reduced intestinal motility at T1 (composite motility grade vs arrival temperature *r*_s_ −0.45, *p* = 0.017) and T2 (cecal motility grade vs arrival temperature *r*_s_ −0.74, *p* < 0.001). Horses with high heart rates, high sweat scores or abnormal demeanour on arrival demonstrated decreased intestinal motility. Salivary cortisol concentrations increased after transportation (mean difference, 95% CI, for T0 vs T1 was 1.66, 1.09−2.53 nmol/L) and were inversely associated with intestinal motility. Signalment and past travel history were not predictive of intestinal motility, but horses with unknown or no prior travel history (mean 12.8, 95%CI 8.2-17.4 nmol/L) had higher cortisol concentrations prior to departure than horses known to have travelled previously (7.9, 5.8−9.9 nmol/L, *p* = 0.023).

**Conclusions:**

These findings suggest that transportation is associated with transient reductions in intestinal motility, particularly during hot ambient conditions and in horses with increased cortisol response. Possible effects of provision of water and food during transport warrant further investigation.

## 1 Introduction

Transportation has been associated with the development of colic in horses associated with gastrointestinal impaction, displacement, obstruction or colitis in epidemiologic (1–3) and in survey (4, 5) studies. Altered intestinal motility has been suggested as contributing to colic (6), and might be related to changes in mucosal transport of water and nutrients (7), or to changes in the gastrointestinal microbiome (8, 9). Decreased gastric emptying and alkaline gastric fluid content, consistent with reflux of duodenal content into the stomach, has been demonstrated in horses transported 12 h overnight (10), suggesting abnormal gastrointestinal motility.

Feeding prior to and during transportation has been recommended to reduce the severity of gastric ulceration subsequent to transportation (10), and makes intuitive sense to many owners. Transport regulations relating to road transport of horses in Europe mandate provision of liquid and, if necessary, feed every 8 h during road transportation.^1^ However, the provision of feed to horses with reduced gastrointestinal motility might predispose to impactions or other abnormalities and hence contribute to adverse outcomes associated with travel. To date, few studies have prospectively evaluated the effect of feeding on gastrointestinal function in transported horses. Percutaneous, transabdominal ultrasonography is a non-invasive technique that has been used to demonstrate alterations in gastrointestinal motility associated with pasture and stable management (11), medications (12–15), anesthesia (16) and colic surgery (16, 17), but has not been previously applied to the evaluation of possible transport effects on intestinal motility in horses.

Physiological alterations, such as increased heart and respiratory rate, increased rectal temperature, and endocrine changes, including alterations to the hypothalamic, pituitary adrenal (HPA) axis, *β*-endorphin (18–20) and serotonergic responses (21), have been associated with transportation and may be influenced by past travel history, temperament, signalment factors and management strategies prior to travel, and with conditions during the journey, including orientation within the transport vehicle, travel distance and duration, ambient conditions such as temperature and humidity. Cortisol increases after road transportation in horses (18, 22–26) and has been used to characterize the hypothalamic–pituitary stress response in this species. The current study aimed to characterize intestinal motility by non-invasive, ultrasonographic assessment of client-owned horses associated with commercial land transportation of 10 – 12 h duration, and to relate gastrointestinal motility to factors evident prior to or during transportation and to the cortisol response observed with travel. We hypothesized that 1. Commercial road transportation without provision of food or water is associated with transiently decreased intestinal motility, 2. Factors evident prior to or during transportation might predict horses at risk for decreased motility, and 3. Horses with higher salivary cortisol concentrations prior to or following transportation will have greater reduction in intestinal motility.

## 2 Materials and methods

### 2.1 Study design

**Figure 1:**
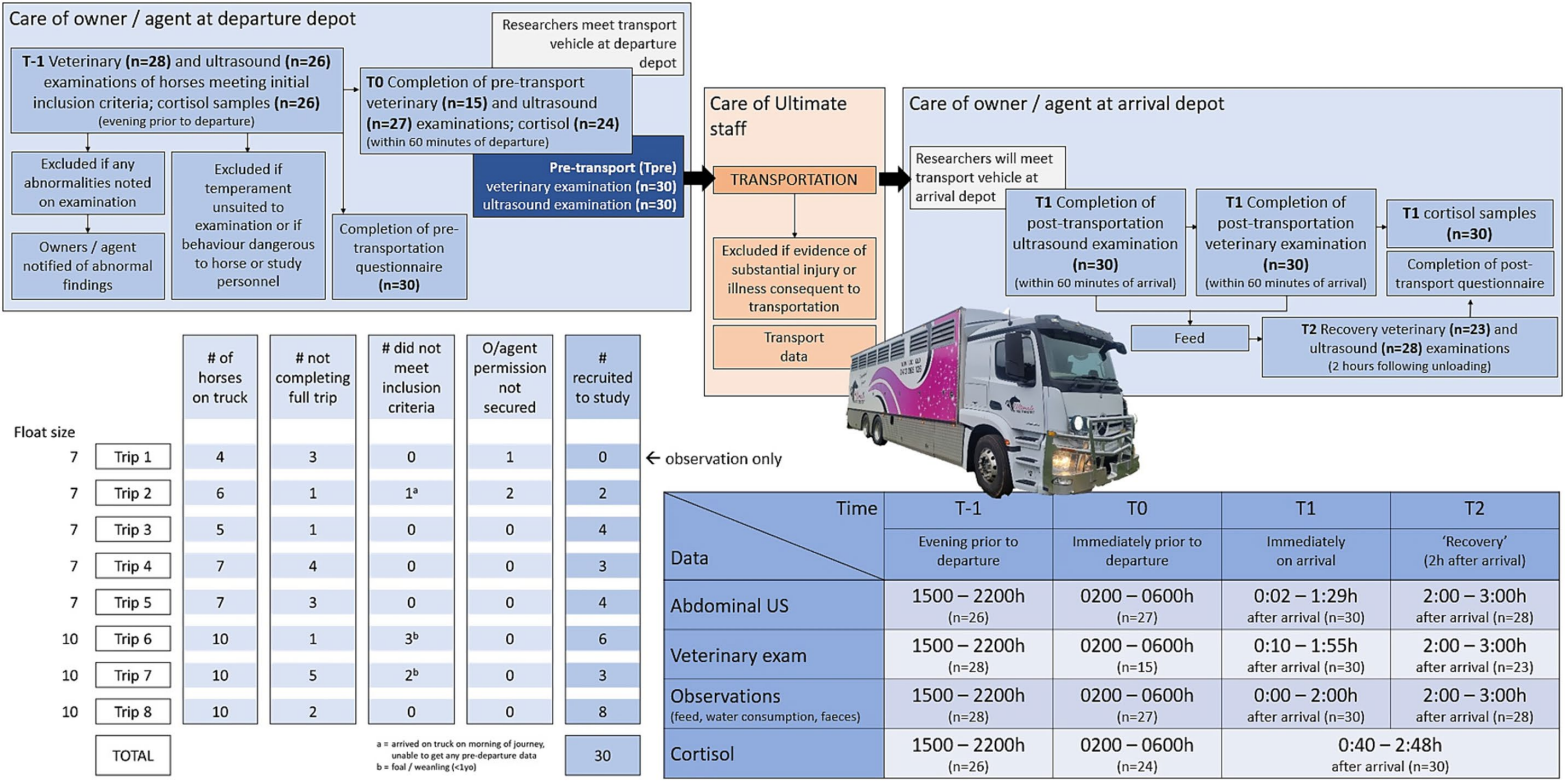
Overview of study design and data collection. Trip 1 was completed by researchers in an observational capacity only (no horses were included in the study); data were collected from horses undergoing seven subsequent trips. The number of horses available for inclusion and sample timing are shown (T-1 and T0 are shown as time of day, T1 and T2 are shown as hh:mm after arrival). Further detail is available in Supplementary information.

This study was conducted as a prospective study using horses completing commercial transportation of 10 to 12 h duration (Figure 1) and was approved by the Charles Sturt University Animal Ethics Committee (approval number A21415). Horses were deemed suitable for inclusion if they were >12 months of age, well-handled, free of substantive injury or illness on veterinary examination, completing a full journey (depot to depot), amenable to and available for veterinary and sonographic evaluation at least once prior to transportation and on arrival. A detailed and standardized data sheet (Supplementary File 1) was completed for each horse enrolled in the study. Veterinary examinations and ultrasonographic assessment (detailed below) were completed, where possible, at the transport depot on the evening prior to transportation (T-1), within 60 min prior to transportation (T0), within 60 min of arrival (T1), and approximately 2 h after arrival (T2). As these were commercial horse movements, researchers were not able to standardize veterinary examination at T-1 or T0 relative to arrival, departure or feeding practices for all horses. As such, times of examination, arrival, feeding and departure were all recorded. Information on horse use, management prior to transportation, reason for transportation (racing, competition, breeding, relocation or other), travel history of each horse, and pre-transport management were obtained from the owner, agent or transport company. Horse age was provided by owner or agent, and confirmed by assessing microchip, brands and/or dentition, as available. Availability and consumption of food and water at T-1, T0 and T2 were recorded; at T1 (off-loading), ultrasonographic and veterinary examinations were completed prior to feeding. Connections (owners, trainers or agents) were asked to document food intake, clinical observations (demeanour, appetite, colicky signs, performance) following transportation using a standardized template (Supplementary File 1) and without knowledge of ultrasonographic findings, to document any adverse effects subsequent to the study and possibly associated with transportation.

### 2.2 Transport details

The study was completed with the support of Ultimate Horse Transport,^2^ departing from and arriving into company depots. Trucks departed at 0500 h for southbound journeys, or at 0400 h for northbound journeys, and arrived at their destination 10 – 12 h later, depending on the number of animals to be dropped off or picked up *en route*, and on local traffic conditions. Horses were offered ad-lib water at all times at the depot, and were fed at least twice overnight or, if resident for longer periods, were additionally fed twice daily. Feeds comprised lucerne hay with additional commercial pelleted feed as needed, or according to owner preferences.

Researchers shadowed the transporter on a preliminary journey (Trip 1, October 2022) to familiarize themselves with depots, and with operations around receival and departure of horses; no data were collected on this initial trip. Journeys were routine (weekly) routes and were included in the current study when two or more horses suitable for inclusion were scheduled to travel, when researchers were available subject to other commitments, and to encompass a range of weather conditions. During transportation horses were managed at the discretion of Ultimate staff, and were not fed or watered in the transport vehicle. Horses were restrained in single bays facing forwards or sideways. Route details and meteorologic information were recorded by researchers using available weather data^3^ and the environment within the transport vehicle was monitored continuously using a weather tracker (Kestrel: 4000 Pocket Weather Tracker, Nielsen-Kellerman, Boothwyn, Pennsylvania). Transport route, distance, duration and driving speed were recorded using a Garmin Edge 540 Cycling Computer (Garmin Australasia Pty Ltd., Marsden Park, NSW) placed next to the weather meter. Loading was videoed and information on loading events was recorded based on researcher observation. Events during transportation were recorded based on driver anamnesis. Horses were unloaded and information on horse demeanour and a sweat score [adapted from Zeyner et al. (27)] were recorded on arrival. Water was available on arrival, and feed was provided immediately following completion of initial sonographic assessment and veterinary examination at T1.

### 2.3 Veterinary examination

Veterinary examinations were performed by researchers (all veterinarians with equine clinical experience and advanced qualifications), assisted by veterinary and equine science students, and were recorded on each horse’s data collection template (Supplementary File 1). Body condition score (BCS) and size (height, girth and shoulder width) were recorded using a measuring stick and measuring tape. Body weight was estimated from girth measurements, and size measurements were converted into a single ordinal size category (extra large, large, medium, small).

Physical examination included inspection of each horse for ocular or nasal discharge or injury, assessment of demeanour, rate and character of respiration, bilateral cardiothoracic and abdominal auscultation, evaluation of circulatory integrity (mucous membrane color and refill, jugular fill, peripheral pulses), and rectal temperature. Abdominal auscultation was performed at the upper and lower quadrants bilaterally, with a subjective grading assigned to each quadrant based on the intensity of borborygmi (0 = silent, no motility heard during 30 s, 1 = less than normal motility, 2 = normal motility, 3 = increased motility) and, in the upper right quadrant, the frequency of ileocecal sounds was recorded as previously described (14, 28).

### 2.4 Characterization of gastrointestinal motility

Ultrasonographic assessment was completed as previously described (15), using a portable, handheld ultrasound probe (GE VScan Air CL, GE HealthCare, Mascot, NSW). Briefly, images were obtained from four acoustic windows to evaluate the duodenum (DUOD, 16th or 17th intercostal space, adjacent to the cranial pole of the right kidney), caecum (CAEC, along the lateral cecal band, ventral to the costochondral junction), colon (COLN, ventral midline or paramedian longitudinal orientation) and jejunum (JEJM, right (or left) inguinal region). Once optimized, 60s video clips of the region of interest were saved using unique, randomly generated 4-digit codes, pre-assigned for each examination. Random identification codes were generated using a standard computer spreadsheet software (Microsoft Excel, Version 2,560, 2025). De-identified clips were independently assessed as previously described (15) by three researchers with specialist qualifications in diagnostic imaging (AC) or equine internal medicine (FF, SLR). For DUOD recordings, researchers counted the number of concentric duodenal contractions. Motility was subjectively graded in each acoustic window, as described previously (15). For each clip, results from the three independent evaluations were combined by taking the mode response. Where discordant results were recorded (defined as the number of DUOD contractions differing by >2 or grade assigned differing by >1), clips and comments were reviewed to achieve a consensus result prior to decoding based on consideration of the character of intestinal movement observed and careful differentiation of patient or probe movement from intestinal contraction.

### 2.5 Cortisol determination

Saliva samples were collected for cortisol determination the evening prior to departure (T-1), immediately prior to loading on the morning of transport (T0) and within 2 h of arrival (between T1 and T2 examinations, subsequently deemed to be T1). Sampling was performed as previously described (29). Briefly, the horse’s head was cradled in one arm and a cotton gauze swab held in Doyen forceps was passed through the diastema between the incisors and premolar teeth. The gauze was placed on the horse’s tongue, and the horse allowed to ‘chew’ for 1 min. On removal from the mouth, the swab was placed in a 20 mL syringe, and the plunger was used to compress the gauze and express saliva (typically ~1 mL) into duplicate Eppendorf containers. Samples were placed on ice and stored at -12°C for up to 24 h prior to storage at -80°C. Saliva cortisol concentration was determined in duplicate using a commercial ELISA kit (Arbor Assay, MI). The limit of detection was 0.12 nmol/L, and intra-assay coefficient of variation was 12%, calculated as SD/mean x 100 from all duplicate samples, as previously described (30).

### 2.6 Statistical methods

The 30 horses included in the current study were a convenience sample based on commercial movements. Power analysis based on previous findings (15) suggested eight horses were sufficient to discriminate a mean difference of two duodenal contractions in the 60s observation window with *α* = 0.05 and power of 0.9, assuming a standard deviation of 1. Additional horses (*n* = 30) were recruited, as available, to enable exploratory assessment of multiple explanatory variables. An overview of explanatory and outcome variables is provided in Supplementary Table S1.

All results were explored initially with descriptive statistics, and continuous data were tested for normality by the Shapiro-Wilks test. Descriptive data relating to subjective observations were grouped and analyzed as categorical or ordinal variables (per data sheet, Supplementary Table S1) using non-parametric methods. The effect of transport on the number of duodenal contractions, the number of ileocecal sounds, and salivary cortisol concentrations was assessed by linear effects model and post-hoc differences were determined by Tukey test. Categorical and non-normal continuous data resistant to transformation were analyzed by Friedman’s test and subsequently by Dunn’s test for pairwise multiple comparisons. In order to provide a complete data set for pre-transportation US assessment, motility results from T-1 and T0 were combined such that ‘pre-transport’ values (Tpre) were derived from the single available result, or from the greater value obtained at T-1 or T0. Associations between outcome variables (measures of intestinal motility and salivary cortisol concentrations) and explanatory variables were explored by univariable analyses: relationships between continuous explanatory and outcome variables were assessed by Pearson correlation if normally distributed or by Spearman correlation; combinations of continuous and categorical variables were explored by Spearman correlation, with effect sizes characterized by unpaired t-test or analysis of variance, or equivalent non-parametric test, as appropriate; effects of categorical variables on categorical outcome data were assessed by Spearman correlation.

## 3 Results

### 3.1 Transport details

The study was completed following horses on seven trips, four southbound and three northbound (Supplementary File 2). Travel duration ranged from 9:47 to 13:15 h, with distances covered between 703 and 1,108 km. Temperatures ranged from a minimum of 10.5°C prior to trip 7, to a maximum of 37.7°C enroute during trip 8. Horses were not offered feed or water during transport, and were restrained in single bays facing forwards or sideways. The majority (*n* = 23) of horses loaded well, six required moderate encouragement to load, and one loaded poorly. Drivers reported at all horses traveled well, and each trip was completed without unexpected incident or delay.

### 3.2 Animals

Thirty horses of mean age 8.4 years (range 18 months to 20 years) and mean weight 493 kg (range 230 to 814 kg) were recruited for the current study. The study population comprised 17 geldings, one entire male, and 12 females, ranging in body condition score from 2 to 5. There were 13 Quarter Horses, six Thoroughbreds, two Standardbreds and nine other breeds (four ponies, three Warmbloods, one Australian Stock Horse, and one Shire-cross).

### 3.3 Veterinary examination

Pre-departure findings are summarized in Supplementary File 3. Horse and researcher availability were managed such that each horse was examined on at least one occasion (T-1 and/or T0) prior to transport. Consistent with inclusion criteria, no horse demonstrated substantive abnormalities on clinical examination prior to transportation at either timepoint.

**Figure 2:**
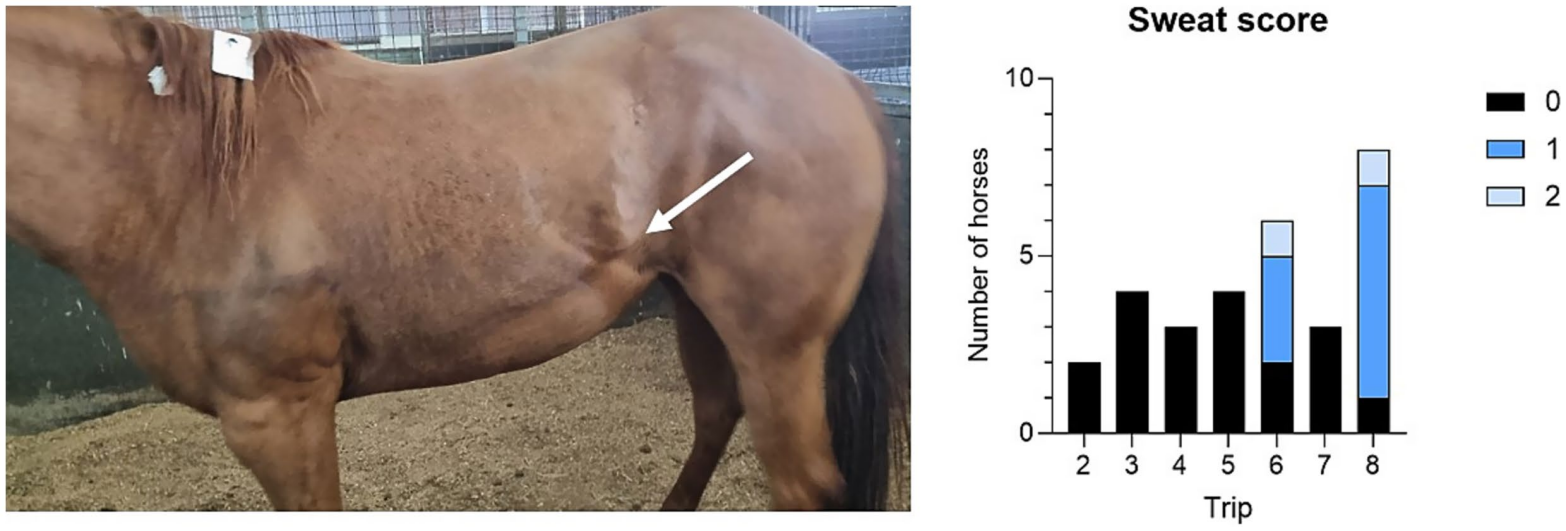
H29 on arrival (left) showing sweat score 2; she is also splinting her abdominal musculature (‘tucked up’), evidenced by muscular line extending from her flank toward her thorax (arrow). Sweat scores on arrival are shown (right) for all horses.

Sweat scores were recorded immediately on off-loading; only horses on trips 6 and 8 were visibly sweating (Figure 2), with four (of six) and seven (of eight) horses scored at 1 or 2 on arrival. Veterinary examination on arrival (T1) was completed for all 30 horses between 10 min and 1:55 h after arrival. Minor increases in heart rate were noted at T1 and persisted at T2 (Supplementary File 3). There were no abnormalities noted on cardiac auscultation for any horse. Jugular refill and peripheral perfusion were considered normal in all horses. Rectal temperature and respiratory rate increased after travel, but were not significantly different to pre-transport values (Supplementary File 3). Gastrointestinal borborygmi included gas sounds on auscultation and/or percussion in four horses, and was significantly reduced in all four quadrants relative to pre-transport findings (Supplementary File 3). Other minor abnormalities were noted on examination of 12 horses (Supplementary File 3).

Veterinary assessment or partial assessment was recorded for 23 horses at T2. Abnormal findings evident at T1 were largely resolved or unchanged (ie. none had progressed). Heart rate was still mildly elevated relative to pre-transport values, and all but two horses had normal mucous membranes. Abnormalities were not appreciated on thoracic auscultation of any horse. Borborygmi remained decreased at T2 in the right ventral quadrant and based on composite score (Supplementary File 3). One horse still had gas on abdominal auscultation. The majority (22 of 28 horses) were observed to eat readily on arrival and most (22 of 28) drank readily after off-loading; observations were not recorded for two horses. No adverse health outcomes were recorded by owners or reported to the transport company subsequent to horse movements included in the current study.

### 3.4 Sonographic assessment of intestinal motility

Ultrasonographic assessment was possible for all 30 horses at T-1 and / or at T0. Results are provided in greater detail in Supplementary File 4. Briefly, examinations were performed between 3:40 and 10:45 pm (T-1), depending on arrival of horses into the departure depot, and prior to transportation (T0) between 1:28 and 6:10 am. Where paired evaluations were completed, there was no difference between results obtained at T-1 in comparison with results obtained at T0 for DUOD (*p* = 0.913, *n* = 23), CAEC (*p* = 0.965, n = 23), JEJM (*p* = 0.518, *n* = 22), or when all motility grades were summed to give a composite score (*p* = 0.051 n = 22). Motility at T-1 was greater than that at T0 in COLN window (median difference 0.5, 98.3%CI 1.0 to 0, *p* = 0.022). The time interval between feeding and sonographic assessment at T-1 or T0 had no effect on the number of duodenal contractions (*p* = 0.697) or DUOD grade (*p* = 0.748), CAEC (*p* = 0.973), COLN (*p* = 0.900), JEJM (*p* = 0.808), or on the composite motility grade (*p* = 0.977).

**Figure 3:**
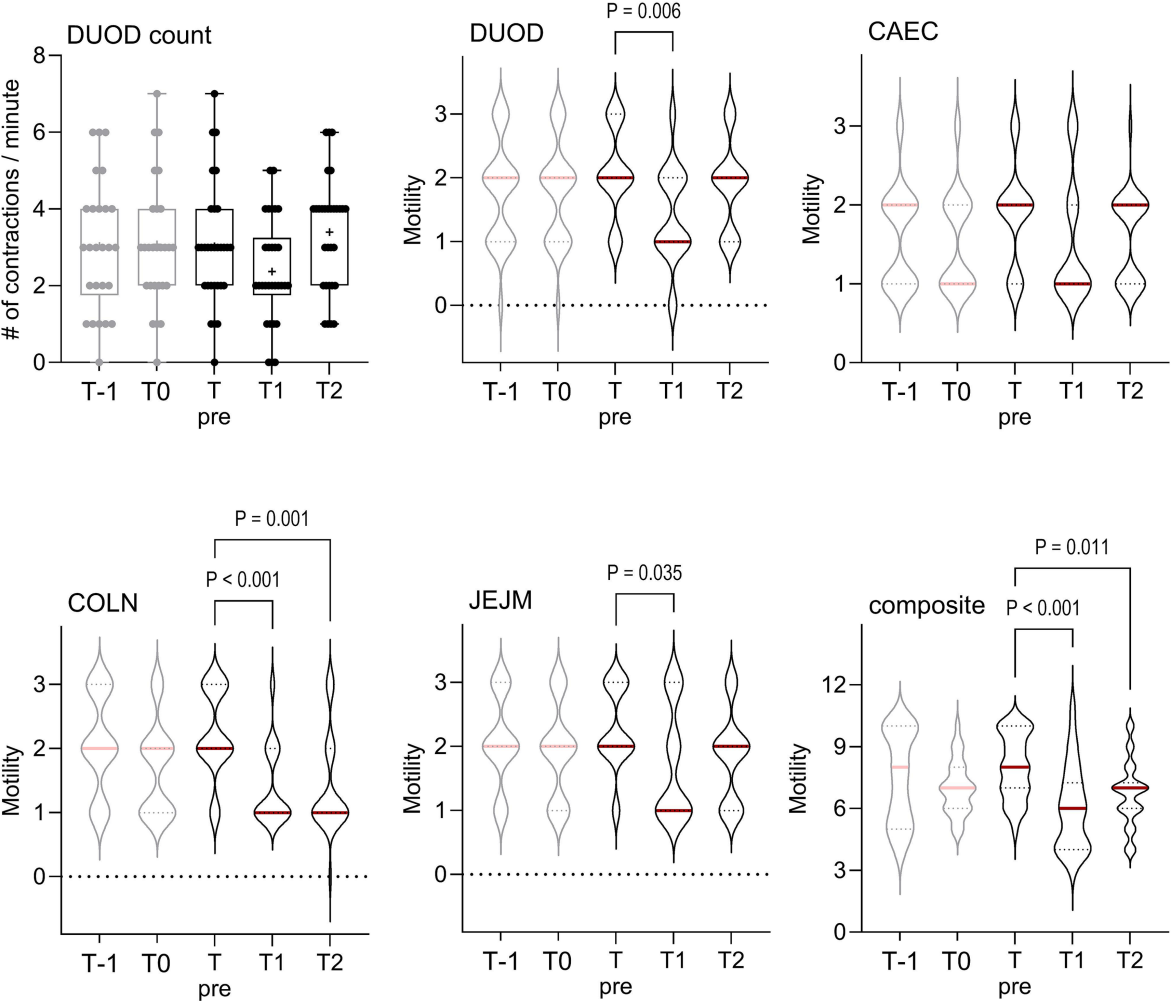
Effects of transportation on sonographic assessments of intestinal motility. The number of duodenal contractions (count) is shown as median (horizontal line), mean (+), quartiles (box), range (whiskers), with all data points shown; motility grades (ordinal data) are shown as violin plots with median (red line) and quartiles (dotted lines) indicated. As graded data were ordinal variables and DUOD count data were nonparametric, all have been analyzed by Friedman test with post-hoc comparisons determined using Dunn’s method. Significant effects were observed for DUOD (*p* = 0.001), CAEC (*p* = 0.026), COLN (*p* < 0.001), JEJM (*p* = 0.005) and the composite motility score (*p* < 0.001), but not for duodenal contractions (*p* = 0.257); significant pairwise comparisons are shown. Results prior to transportation at T-1 and T0 are shown, but not included in analyses. DUOD = nephro-duodenal window, CAEC = caecal window, COLN = ventral colon window, JEJM = jejunal (inguinal) window; # = number.

Sonographic assessment was repeated between 2 min and 1:29 h after off-loading horses at the arrival depot (T1), prior to horses being fed, and again at T2, 1:43 to 4:12 h after arrival. When compared to Tpre values, transportation was associated with decreased motility grades in DUOD (*p* < 0.001), CAEC (*p* = 0.024), COLN (*p* < 0.001) and JEJM (*p* = 0.004) windows, and in the composite motility score (*p* < 0.001), as shown in Figure 3. Results at T2 were equivalent to pre-transport values for all windows except COLN and composite grades.

**Figure 4:**
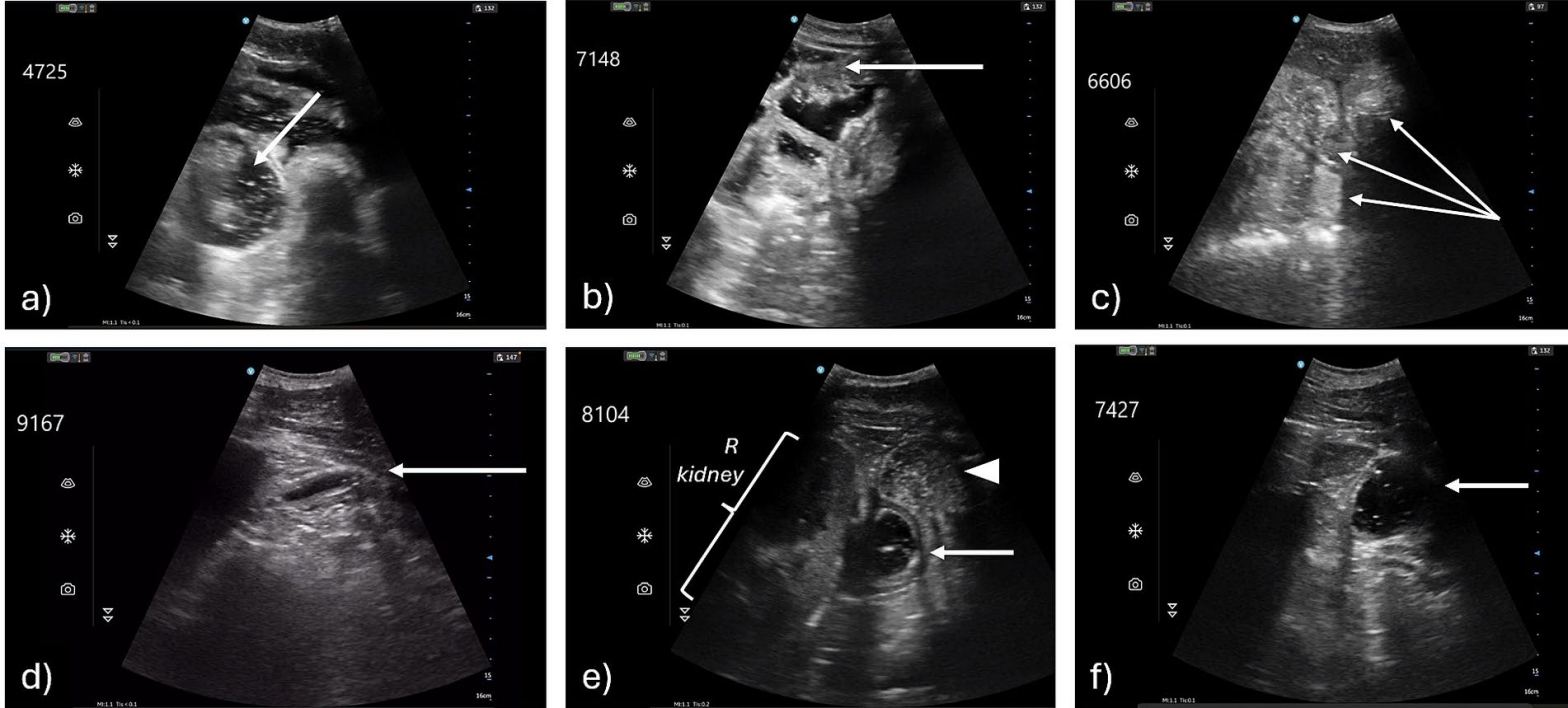
Representative jejunal (top row) and duodenal (bottom) images showing qualitative abnormalities recognized after transportation; all images were obtained at T1. For jejunal images, ventral is to the top of the image; for duodenal images, cranial is to the left of the image. **(a)** H16 – jejunal loops are more prominent and there is increased anechoic content with partitioning of content (arrow), there is normal to increased motility evident in cineloop (Supplementary material); **(b)** H12 – as for recording 4725, sedimentation is more obvious (arrow); **(c)** H23 – mildly distended jejunal loops are evident (arrows) with anechoic content; motility is normal to slightly decreased (Supplementary material); **(d)** H2 – duodenal motility is reduced, duodenum (arrow) remains slightly distended, with anechoic content; **(e)** H12 – there is fluid content in a dilated duodenum and a second viscus noted more superficially and ventrally (arrowhead) containing more echogenic fluid; the right kidney is more evident to the left (dorsal) of frame in this image; **(f)** H16 – there is anechoic fluid in a moderately distended duodenum (arrow).

Qualitative differences were recognized in images obtained after transportation, particularly in the DUOD and JEJM windows (Figure 4; cineloops are provided as Supplementary Files). Qualitative changes in duodenal function were recognized in one horse at T-1, six horses at T1, and one horse at T2. In each instance, evaluators recognized decreased or abnormal contractility and increased, typically anechoic, fluid content in the duodenal lumen. In one case gas shadowing was observed. Qualitative changes in small intestinal function in the JEJM window were recognized in 12 horses at T1, and were characterized as moderately distended, fluid-filled loops with, variably, decreased motility, partitioning or sedimentation of content. Less pronounced changes persisted in two horses in T2 recordings.

### 3.5 Factors associated with intestinal motility after transport

Ambient conditions on the day of transport were significantly associated with sonographic measures of intestinal motility following transportation (Table 1), with reduced cecal and composite motility grades at T1 observed in association with high ambient temperatures during the journey and on arrival. This effect was still present, in fact was more pronounced, at T2. Heart rate on arrival (T1) was inversely associated with duodenal motility at this time, and changes identified on arrival (T1) including heart rate, sweat score and demeanour (abnormally quiet or abnormally agitated), were associated with reduced duodenal and cecal motility. There was no direct and consistent association between auscultatory findings and sonographic motility grades but, at T1, auscultation of the left ventral abdomen was positively associated with JEJM motility grade, and auscultation of the right ventral abdomen was positively associated with COLN, JEJM and COMP grades. The composite auscultation grade was positively associated with JEJM and COMP sonographic motility grades at this time. Horses that had spent >1 day at the transport depot were more likely to have decreased sonographic motility on arrival, and the amount of food consumed the night prior to departure was positively associated with the.

**Table 1:** Factors significantly associated with sonographic motility after off-loading from transport (T1) and on recovery (T2, 2 h after arrival).

Outcome variable		Explanatory factor(s)	Significance P=	Effect size
T-1 Intestinal motility				
	DUOD count	T-1 [cortisol]	0.018	r_s_ = −0.469
	DUOD grade	T-1 [cortisol]	0.008	r_s_ = −0.336
	COLN grade	T-1 [cortisol]	0.046	r_s_ = −0.402
	COMP grade	T-1 [cortisol]	0.018	r_s_ = −0.468
T1 Intestinal motility				
	DUOD count	HR at T1	0.021	r = −0.419
	DUOD grade	HR at T1	0.004	r_s_ = −0.506
	CAEC grade	Waypt temp	0.018	r_s_ = −0.435
		Arrival temp	0.048	r_s_ = −0.371
		Time at depot prior to travel	0.016	Depot ≤1d 2 (1–3), depot >1d 1 (1–1)*
	COLN grade	RV ausc at T1	0.012	r_s_ = 0.261
	JEJM grade	LV ausc at T1	0.030	r_s_ = 0.226
		RV ausc at T1	0.019	r_s_ = 0.245
		COMP ausc at T1	0.011	r_s_ = 0.265
	COMP grade	Arrival temp	0.017	r_s_ = −0.448
		RV ausc at T1	0.001	r_s_ = 0.329
		COMP ausc at T1	0.014	r_s_ = 0.256
		Time at depot prior to travel	0.008	Depot ≤1d 7 (6–9), depot >1d 4 (4–6.75)
		Feed overnight prior to travel	0.050	Ate all 7 (5–9), ate ~½ 6 (4–11), ate <½ 4 (3–4)*
T2 Intestinal motility				
	DUOD count	Sweat score at T1	0.068	r_s_ = −0.350
		Demeanour at T1	0.020	Normal 3.88 (3.11–4.65), abnormal 2.54 (1.73–3.36)**
	CAEC grade	Waypt temp	<0.001	r_s_ = −0.782
		Arrival temp	<0.001	r_s_ = −0.740
		Waypt humidity	0.002	r_s_ = 0.630
		Arrival humidity	<0.001	r_s_ = 0.566
		Sweat score at T1	0.001	r_s_ = −0.579
		Demeanour at T1	0.004	Normal 2 (1.5–2), abnormal 1 (1–1)*
		T1 [cortisol]	0.007	r_s_ = −0.497
	JEJM grade	T0 [cortisol]	0.041	r_s_ = −0.439
	COMP grade	Sweat score at T1	0.013	r_s_ = −0.462

*Median (IQR); **Mean (95% CI). DUOD, duodenum; CAEC, caecum; COLN, ventral colon; JEJM, jejunum; COMP grade, composite (summed) motility score; waypt temp, waypoint temperature (weather data from mid-point of trip); arrival temp, ambient temperate on arrival; ausc, auscultation; HR, heart rate; RV, right ventral abdomen; LV, left ventral abdomen; COMP, composite auscultation score; composite (sum of motility scores); IQR, interquartile range (non-parametric data); 95% CI, 95% confidence interval (parametric data). Interactions between continuous explanatory data with continuous outcomes data were explored by Pearson (parametric) or Spearman (non-parametric) correlation; effects of categorical explanatory factors on continuous data were explored by analysis of variance or t-test (or non-parametric equivalent, as appropriate), and effects of categorical explanatory data on categorical outcomes were evaluated using Spearman correlation.

### 3.6 COMP motility grade on arrival

Sonographic findings at T1 or T2 were not readily associated with appetite nor fecal output on arrival. Sonographic measures of motility prior to transportation (T-1 or T0) were not associated with results following transportation, nor was motility at T1 associated with motility at T2. Signalment factors including use, level of exercise and past travel history were not associated with sonographic motility at any time during the current study.

### 3.7 Cortisol response to transportation

**Figure 5:**
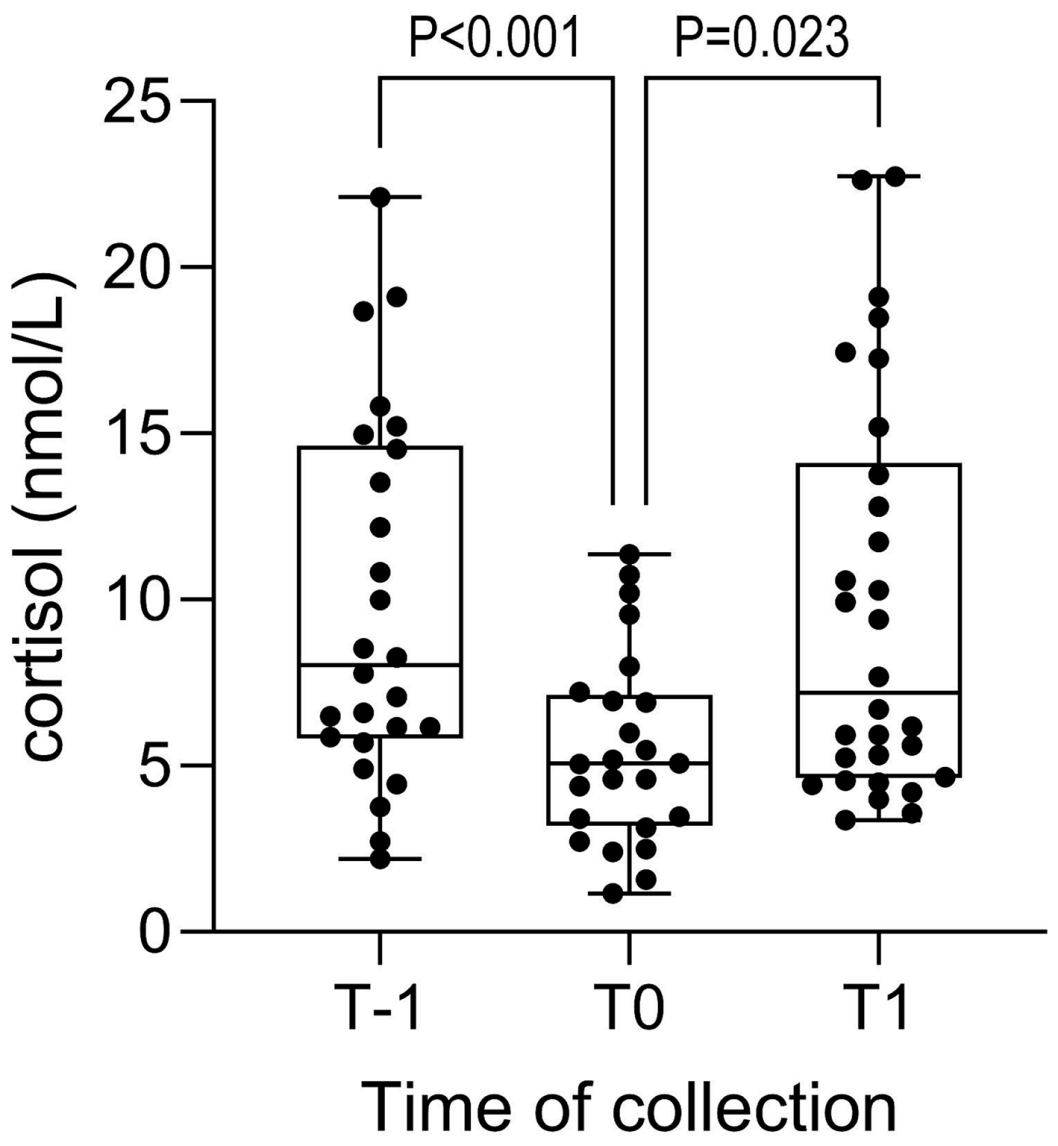
Salivary cortisol concentrations the evening prior to travel (T-1), immediately prior to loading (T0) and on arrival (T1). Data are shown as median (horizontal line), quartiles (boxes) and range (whiskers), with all data points shown. Data were non-normal and transformed for analysis by linear effects model. A significant time effect is evident (p < 0.001), with results of post-hoc pairwise comparisons (Tukey test) shown.

Transportation was associated with increased median salivary cortisol concentrations in samples collected between 40 min and 2:48 h after transportation (T1, Figure 5), relative to values obtained prior to transportation (T0). Factors associated with salivary cortisol concentrations at T-1 and T1 are shown in Table 2. None of the factors evaluated was associated with salivary cortisol concentrations at T0. Horses with no previous experience of travel, or with unknown travel histories, had significantly higher cortisol concentrations at T-1 than was observed for horses that had traveled occasionally or frequently. Higher temperatures during travel (waypoint temperature) and on arrival, and lower humidity at waypoint and on arrival, were associated with higher cortisol concentrations at T1. Cortisol at T1 was significantly and inversely associated with distance, duration of travel and arrival time. Salivary cortisol concentrations increased as the interval between arrival and sampling increased, and a similar effect was observed for time of sampling. Cortisol responses varied with trip, with horses on trip 8 showing greater cortisol responses at all sampling times.

**Table 2:** Factors significantly associated with saliva cortisol concentrations the night prior to transportation (T-1) and after transport (T1).

Outcome variable	Explanatory factor(s)	Significance P=	Effect size
T-1 Cortisol concentration			
	Travel history	0.029	Nil/unknown 12.8 (8.2–17.4), occ/freq 7.9 (5.8–9.9) nmol/L**
T1 Cortisol concentration			
	Waypoint temperature	<0.001	r_s_ = 0.572
	Arrival temperature	0.002	r_s_ = 0.535
	Waypoint humidity	<0.001	r_s_ = −0.585
	Arrival humidity	0.001	r_s_ = −0.569
	Distance traveled	0.002	r_s_ = −0.533
	Duration of travel	0.004	r_s_ = −0.514
	Time of arrival	0.011	r_s_ = −0.457
	Time of sampling	0.018	r_s_ = −0.428
	Interval btw arrival & sampling	0.002	r_s_ = 0.535
	Trip	<0.001	Trip 8 16.2 (13.0–19.0), other trips 5.8 (4.5–9.5) nmol/L*
	Sweat score	<0.001	r_s_ = 0.653
	Abnormal demeanour	0.005	r_s_ = 0.497

*Median (IQR); **Mean (95% CI). occ, occasional; freq, frequent; btw, between; IQR, interquartile range (non-parametric data); 95% CI, 95% confidence interval (parametric data). Interactions between continuous explanatory data with continuous outcomes data were explored by Pearson (parametric) or Spearman (non-parametric) correlation; effects of categorical explanatory factors on continuous data were explored by analysis of variance or t-test (or non-parametric equivalent, as appropriate), and effects of categorical explanatory data on categorical outcomes were evaluated using Spearman correlation. No significant interactions were observed for salivary cortisol concentrations immediately prior to departure (T0).

## 4 Discussion

This study demonstrated decreased intestinal motility associated with road transportation, and prospectively evaluated environmental and horse factors that might be associated with the magnitude of observed change. Using four acoustic windows on the right side of the horse, sonographic measures of intestinal motility were reduced after transportation in the duodenal (DUOD), jejunal (JEJM), and colon (COLN) windows, and when all scores were combined (COMP). Horses in the current study demonstrated no clinically apparent adverse effects and recovered quickly after transportation, but sonographic assessment also identified qualitative changes consistent with small intestinal dysmotility. As part of veterinary assessment of transported horses, such changes might be monitored on arrival and inform management or husbandry decisions, such as ongoing monitoring, feeding, or performance expectations. These changes were easily recognized and were quite different to normal motility patterns. Taken together with previous studies that reported decreased gastric emptying and reflux of duodenal content into the stomach in transported horses (10), these findings suggest that transportation might be associated with loss of normal aboral movement of intestinal contents.

In the current study, ambient temperature likely exacerbated the observed effect on motility. Heat stress during transportation has been recognized as a welfare issue for horses (31), and heat stroke has been identified in owner surveys of health problems associated with transportation in this species (32). Variations in hormonal and autonomic regulation during road transport of horses in tropical conditions have been linked to ambient conditions (33) but, to the authors’ knowledge, this is the first study to demonstrate an association between ambient temperature and intestinal function in transported horses. On arrival, an association was evident between reduced motility and increased sweat score, abnormal demeanour and increased heart rate, suggesting that these factors, evident on physical examination, might predict horses at increased risk of adverse gastrointestinal sequelae to transportation. The inverse relationship observed between heart rate and motility is consistent with a switch to sympathetic dominance as part of the sympathetic-adrenal medullary stress response in anticipation of or response to transportation and off-loading to a novel environment. Sweat score and demeanour on arrival were also associated with reduced motility at T2, suggesting that visibly affected horses might fail to recover as well as other horses following transport. Sonographic evaluation of intestinal motility might provide complementary information to that obtained from auscultation. The grade of intestinal sounds (borborygmi) on abdominal auscultation was positively associated with some sonographic measures of intestinal motility, suggesting that the reciprocal finding (decreased gastrointestinal sounds) might predict decreased motility, although sonographic and audible measures of motility were not strongly associated at any time point or, anecdotally, when researchers were able to perform both assessments concurrently. Similar observations have been made in human studies, where sonographic evaluation has been interpreted as superior to auscultation for assessment of intestinal function in people (34, 35).

Horses that had been at the transport depot for a protracted time, or that ate little overnight prior to transport, were more likely to have decreased intestinal motility on sonographic evaluation at off-loading. However, to the extent evaluated in the current study, factors identified prior to transportation, including prior transport history and signalment factors such as breed, size or intended use, were not predictive of alterations in intestinal motility, before or after the journey. Prior to travel (T-1, T0), there was no clear association between intestinal motility and other observations, such as feed consumption, water intake, fecal output or physical exam findings. Despite the absence of external evidence of physiologic ‘stress’ response, sonographic measures of intestinal motility at T-1 were inversely associated with salivary cortisol concentrations, suggesting that intestinal function was adversely affected by the hypothalamic–pituitary stress response. In the time frame available for assessment in the current study, sonographic results at off-loading or 2 h after arrival were not predictive of food intake or fecal output following transport, and were only weakly associated with cortisol responses.

Cortisol concentrations in saliva samples obtained at all time points in the current study were well above those reported in resting horses (29), and consistent with previous studies on transported horses. Values obtained at T-1 and T1 were increased relative to data from T0, demonstrating reversal of the expected diurnal variation [values should be highest in the morning (29)]. Horses with prior travel experience had slightly lower cortisol values at T-1 than was observed for horses that had not traveled before or where travel history was unknown, suggesting that prior experience mitigated the stress response associated with travel. Hot weather was associated with the highest cortisol values, but no consistent association was identified with signalment, body size, or factors evident on clinical examination. Surprisingly, cortisol concentrations increased as the time interval between arrival and sampling increased, and with samples derived later in the day. While these findings might be confounded by other factors, in the current study, horses were off-loaded into an environment with which they had no prior experience. By following horses in a commercial transport environment, the current study differs from previous results evaluating horses transported from and returning to familiar environments (29, 36, 37), and might suggest that, rather than ameliorating endocrine responses to transportation, arrival into a novel environment may be an additional stressor for horses.

Sonographic assessment of intestinal motility was readily completed in a field setting with portable hand-held ultrasound transducers. Horses in the current study spanned a range of handling experience and temperaments but readily accepted the procedure with minimal restraint. Sonographic evaluation proved effective to further characterize horse health beyond what is possible with veterinary examination alone, demonstrating health conditions that might have influenced wellbeing during transport (pregnancy, abnormal abdominal fluid, as documented in Supplementary File 4). Although not pursued in the current study, ultrasonographic evaluation might include assessment of gastric size and content (15, 17) or pulmonary changes (38).

### 4.1 Limitations

As might be expected in commercial settings, trips included in the current study were opportunistic, of slightly different durations, and difficult to schedule. In some weeks an unseasonably low number of horses was booked for travel, meaning that some journeys were canceled due to insufficient numbers of horses meeting inclusion criteria and with owner permission for participation in the study. As these were commercial horse movements, researchers did not influence management practices of horses prior to departure, during transportation or on arrival. To facilitate rapid acquisition of ultrasonographic images and veterinary examination data immediately on arrival, sonographic assessment was restricted to the right side of the horse. Data analysis included available results at T-1 and T0 but, in some circumstances, horses were not available for assessment at both time points. To enable repeated measures analyses without missing data points, results at T-1 and T0 were pooled. Although the validity of data handling in this way might be contested, the combined pre-transport (Tpre) result was considered to reflect the best available measure of predictive variables, and direct comparison of available results suggested there was no significant difference between values obtained at T-1 and T0. However, diurnal variation and the effect of management practices, particularly the time since feeding, might be expected to influence values of interest, including intestinal motility. The failure to identify any such effect might suggest that factors in the transport environment influenced normal homeostatic mechanisms.

Veterinary examination at T1 was completed between 10 min and 1:55 h after arrival, and sonographic assessment was completed between 2 min and 1:29 h after arrival. Due to logistic constraints in a commercial setting, these were much wider windows than was planned and this allowed variable recovery prior to examination for horses. Continuous data recoding with ‘smart textiles’, telemetry ECG or electronic sensors (14) would be a pragmatic response to this challenge in future studies. However, evaluation of horses at T2 yielded more significant differences and greater effect size than was apparent for T1 data, suggesting that abnormalities persisting in horses after transportation and off-loading might be of more value than their immediate responses.

The apparent lack of effect on intestinal motility of explanatory factors such as travel history, signalment, management and physical examination findings prior to transportation should be interpreted cautiously given incomplete data collection, the relatively small number and the heterogenous population of horses included in the current study. Behavior during transport was based on driver anamnesis rather than direct observation. Given these limitations, multivariate analysis was deemed inappropriate and some apparent associations might reflect interactions between factors that could not be reliably evaluated. For example, counter-intuitive associations between relative humidity and journey duration might be confounded by the strong observed effect associated with ambient temperature. Horses in the current population were well handled, loaded easily and traveled well. Our findings, therefore, may not be transferrable to less well-handled horses. Finally, as an observational study of commercial horse movements, the current study could not evaluate the effects of provision of feed or water during the journey, and we were unable to include a control group of horses fasted for an equivalent period of time without transportation. In a commercial transport setting, we were unable to collect reliable measures of urine volume or fecal output and consistency. Future studies might include more rigorous evaluation of fecal output and urine production as measures of physiologic response to transport conditions.

## 5 Conclusion

In the current study, transport without provision of food or water was associated with quantitative and qualitative adverse effects on intestinal motility, confirming previous observations relating to gastric emptying (10). High ambient temperatures potentiated this effect and were also associated with increased salivary cortisol concentrations. Until we have further evidence upon which to base feeding practices during and subsequent to transportation, horses should be provided with food and water on arrival and be evaluated carefully after transport, as some characteristics readily identifiable on arrival (sweat score, demeanour and heart rate) might identify horses at increased risk for decreased intestinal motility or delayed recovery. Other abnormalities evident on physical examination in the current study were relatively uncommon, of minor severity and variably involved the gastrointestinal tract, respiratory tract or minor injury to limbs or body surfaces. Future studies should ascertain whether the provision of food and/or water during travel might ameliorate the detrimental effects on intestinal motility observed in the current study or, conversely, whether fasting during transportation might protect horses from colic subsequent to the observed motility disorders. Conversely, ingestion of feed, particularly in the absence of adequate water intake during travel, might predispose to impaction or colic if the observed changes are a response to the transport environment. The experimental methodology used in the current study is well suited to addressing research questions designed to further characterize gastrointestinal responses to transportation.

## Data Availability

The original contributions presented in the study are included in the article/Supplementary material, further inquiries can be directed to the corresponding author/s.

## Footnotes

^1^Council Regulation (EC) No. 1/2005 on the protection of animals during transport and related operations and amending Directives 64/432/EEC and 93/119/EC and Regulation (EC) No. 1255/97. Chapter V *Watering and feeding interval, journal times and resting periods,* regulation 1.4(c), page 25. https://eur-lex.europa.eu/legal-content/EN/TXT/PDF/?uri=CELEX:32005R0001, accessed 25/06/15.

^2^1,236 Tooma Rd., Burra, NSW; ultimatehorsetransport.net.au

^3^ http://www.bom.gov.au/
