# Supplementary material for: Road transportation is associated with decreased intestinal motility in horses
Source: Front Vet Sci. 2025 Aug 18;12:1647236. doi: 10.3389/fvets.2025.1647236 (PMC12401009; doi:10.3389/fvets.2025.1647236)
Supplement: Supplementary file 1 [file Table_1.docx]

**Altered gastrointestinal motility as a risk-factor for colic associated with transportation of horses**

**Pre-transportation information**

Owner / agent details [*de-identified prior to analysis*]

Name of horse _______________________________ Name: ________________________________

Breed Thoroughbred Other _____________ Contact details: _________________________

Age ____________ years (DOB: ________)

Gender Male / Male castrate / Female Brand:

Use / activity: racing / breeding / performance / other (specify: _________________________________________ )

Training / level of activity: nil / minimum / moderate / competing

Frequency: nil / monthly or less / weekly / daily

Intensity / duration: low / moderate / high (details: _________________________________________ )

Racing / competing: yes / no Last race: __________________________________________________

Current form / health: poor / recovering / good (no problems)

If poor / recovering, provide details:

Current medication (specify):

**MM’s**

**Prior travel history**

Transported previously: yes / no

Number of times: few / moderate / many / unknown

(1 or 2 trips) (>10 trips)

Previous history of adverse events (illness / injury) associated with transportation: yes / no / unknown

Details: __________________________________________________________________________________________

________________________________________________________________________________________________

________________________________________________________________________________________________

**Feed management prior to transportation**

Diet: Grain (type/s: _________________________________________________________ )

Pellets / commercial (specify: _____________________________________________ )

Hay (type/s: ___________________________________________________________ )

Pasture access: yes / no (type/s and frequency / duration: ________________________________ )

Supplements (type/s: ___________________________________________________ )

Other:

**Management at depot** *(routine feeding and management practices in note book – only record variations)*

Location prior to transportation:

Arrival at depot:

Transport time to depot (approx.):

Feed management at the depot: standard (Ultimate / Goldners / other) changed: _____________

Last fed:

**Pre-departure veterinary examination (T-1)** Time: _______________ Initials: ___________

HR __________ bpm MM colour and CRT: _____________________________

RR __________ bpm Peripheral perfusion, jugular refill: ___________________

Rectal Cardiac auscultation: _____________________________

Temperature _________ ^o^C Pulmonary auscultation: ___________________________

Abdominal auscultation:

0 absent

+ decreased

++ normal

+++ increased

Demeanour: ICS ____ / 2 minutes

LD RD

LV RV

Dull QAR BAR Excited

Other (specify)

Injuries or other abnormalities:

Body condition score:


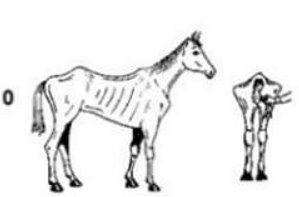

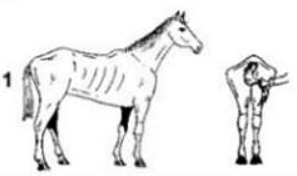

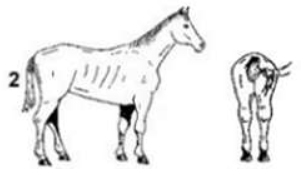


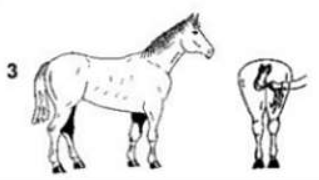

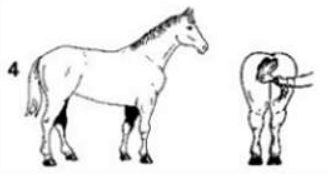

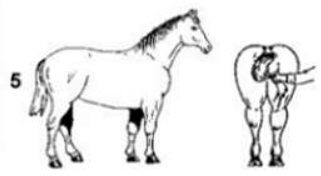


(adapted from Carroll C.L. and Huntington P.J., Body Condition Scoring and Weight Estimation of Horses)

Size and weight: height: _________ cm; girth: _________ cm; body weight (tape): _________ kg; shoulder: _________ cm


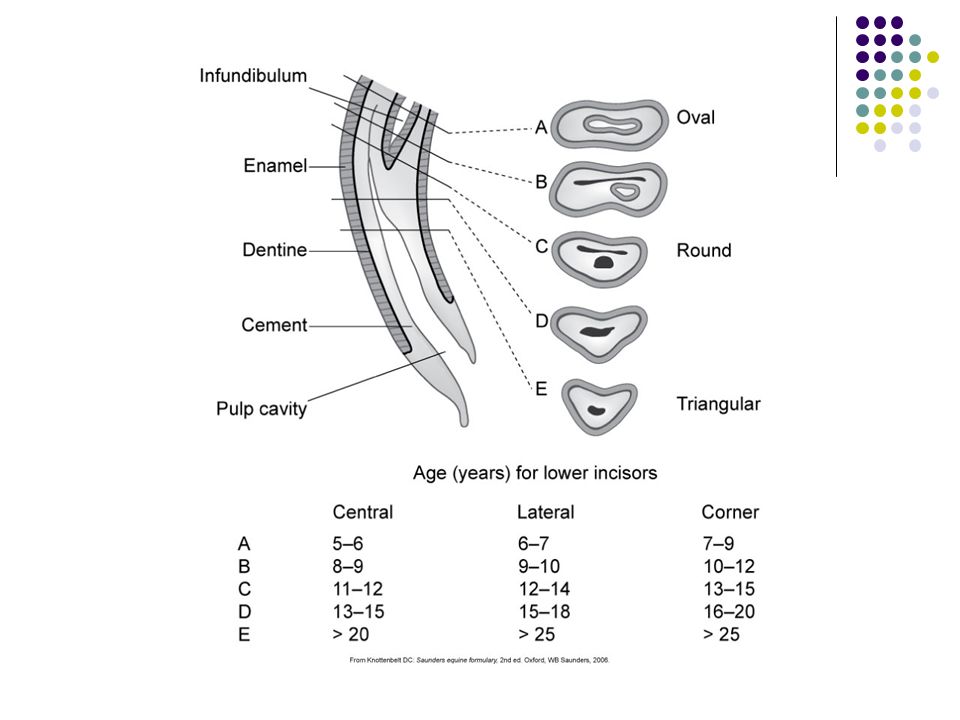
Age (dentition): ________________________________________________________________________________


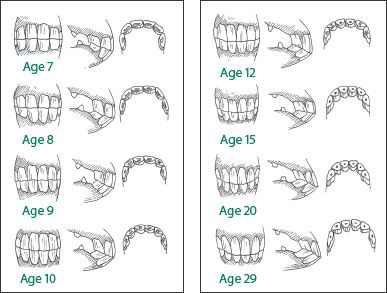


**Pre-departure abdominal ultrasound examination (T-1)**

Date: _________________ Time: ______________________ Performed by: _______________________

| Duodenal motility | Caecal motility | Jejunal motility | Ventral colon motility |
| --- | --- | --- | --- |
| *Visualized adjacent to R kidney* | *R lateral taenial band of caecum, ventral to costochondral junction* | *Image R or L inguinal region, or ventral abdomen* | *Image ventral abdomen, to right of midline* |
| Count number of circular contractions during 60s cineloop: | Grade movement:  0 = there is no movement  1 = there is infrequent (<2) mixing mvt evident  2 = there are 1 or 2 contractions (mvt away from probe / abdo wall) &/or frequent mixing contractions  3 = there are frequent contractions (>2) or continuous mixing movements | Grade visualization:  0 = no jejunal loops viz  + = 1 or 2 loops intermittently visible  ++ = 1 or 2 loops visible for most or all of video (60s)  +++ = multiple loops visualized  Grade movement:  0 = there is no movement  1 = mvt of ingesta <50% of recording  2 = mvt of ingesta >50% of recording  3 = near continuous motility  Sediment (yes/no) | Grade movement:  0 = there is no movement  1 = mvt of ingesta <50% of recording  2 = mvt of ingesta >50% of recording  3 = near continuous motility |
| File saved as*  DUOD _ _ _ _ _ | CAEC _ _ _ _ _ | JEJM _ _ _ _ _ | COLN _ _ _ _ _ |

* allocate random number from list provided

Saliva sample T-1: Time:


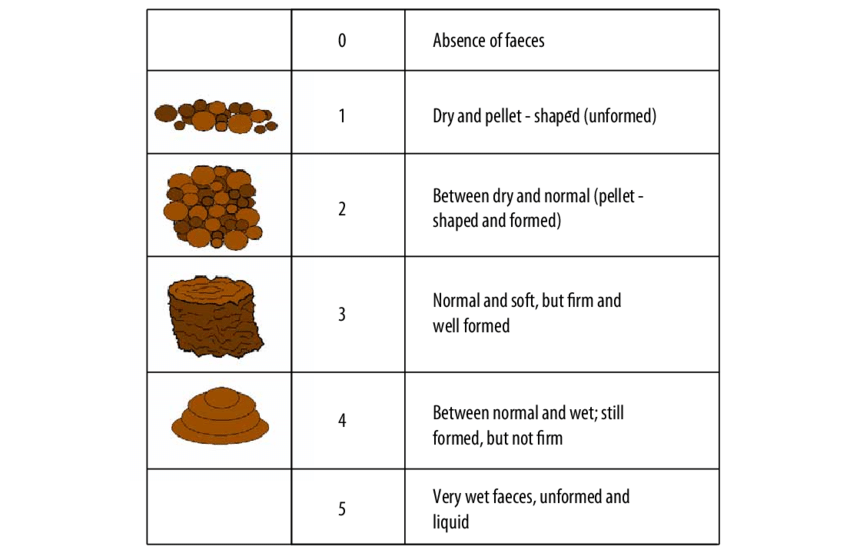


Faecal score T-1: Number of faecal piles in box:

Faecal sample collected 🞎

Sample number: _________________

**Pre-departure veterinary examination (T0)** Time: _______________ Initials: ___________

(if possible)

HR __________ bpm MM colour and CRT: _____________________________

RR __________ bpm Peripheral perfusion, jugular refill: ___________________

Abdominal auscultation:

0 absent

+ decreased

++ normal

+++ increased

Demeanour: ICS ____ / 2 minutes

LD RD

LV RV

Dull QAR BAR Excited

Other (specify)

Feed consumed overnight (circle): All >1/2 <1/2 Little / none

Water consumed overnight (circle): >1/2 bucket ¼ to ½ bucket Almost none Spilled Unable to assess

Feed prior to loading (circle): All >1/2 <1/2 Little / none

**Pre-departure (T0) US examination** (if possible)

Date: _________________ Time: ________________________ Performed by: ___________________

Stomach US: not distended moderate distension marked distension

| Duodenal motility | Caecal motility | Jejunal motility | Ventral colon motility |
| --- | --- | --- | --- |
| *Visualized adjacent to R kidney* | *R lateral taenial band of caecum, ventral to costochondral junction* | *Image R or L inguinal region, or ventral abdomen* | *Image ventral abdomen, to right of midline* |
| Count number of circular contractions during 60s cineloop: | Grade movement:  0 = there is no movement  1 = there is infrequent (<2) mixing mvt evident  2 = there is 1 or 2 contractions (mvt away from probe / abdo wall) &/or frequent mixing contractions  3 = there are frequent contractions (>2) or continuous mixing movements | Grade visualization:  0 = no jejunal loops viz  + = 1 or 2 loops intermittently visible  ++ = 1 or 2 loops visible for most or all of video (60s)  +++ = multiple loops visualized  Grade movement:  0 = there is no movement  1 = mvt of ingesta <50% of recording  2 = mvt of ingesta >50% of recording  3 = near continuous motility  Sediment (yes/no) | Grade movement:  0 = there is no movement  1 = mvt of ingesta <50% of recording  2 = mvt of ingesta >50% of recording  3 = near continuous motility |
| File saved as*  DUOD _ _ _ _ _ | CAEC _ _ _ _ _ | JEJM _ _ _ _ _ | COLN _ _ _ _ _ |

Saliva sample T0: Time:

Faeces in box (o/nte): __________ piles Score: _____________ Sampled? **(T0)**

**Transportation details**

| Loaded: | Well  (minimal or no resistance) | | With moderate encouragement | | Poorly  (multiple attempts – record time taken) | |
| --- | --- | --- | --- | --- | --- | --- |
| On truck prior to departure (or enroute): | | Standing quietly | | Somewhat agitated | | Marked agitation  (vocalising, pawing, other) |

**Departure point: ____________________ Destination: _____________________________**

**Departure time: ____________________ Arrival time: _____________________________**

| Travelled: | Well  (quiet & calm during entire trip) | Mostly well  (agitated at beginning of trip or occasional mild agitation, but mostly calm) | Agitated  (agitated or distressed at beginning of & during trip; abn behaviour more intense or prolonged) | Poor  (pawing, scrambling, vocalising during much of the trip; abn behaviour intense or prolonged) |
| --- | --- | --- | --- | --- |

Observation / events *en route*:

| Demeanour on arrival: | somnolent / obtunded | normal | agitated / excited |
| --- | --- | --- | --- |
| Other or comment: __________________________________________________________________________ | | | |

Feed available during transit: yes / no details: ______________________________ amount consumed:

Water available during transit: yes / no details: ______________________________ amount consumed:

(include amounts offered and consumed, if applicable)

Sweat score on arrival (adapted from Zeyner et al 2014, *J An Phys Nut* 98: 246-250):

| 0 | No visible sweat |
| --- | --- |
| 1 | Focal areas sticky / moist, sticky throat area, flanks darker than normal |
| 2 | Focal areas obviously moist or small white areas of foam, friction surfaces related to tack |
| 3 | Tack leaves clear wet impression on the head, typically with foam or obvious focal wet areas on body / flanks |
| 4 | Throat and flanks completely wet, moist dark wrinkles above eyes, sweat / foam between hindlimbs |
| 5 | Horses additionally dripping fluid above the eyes and/or under the belly |


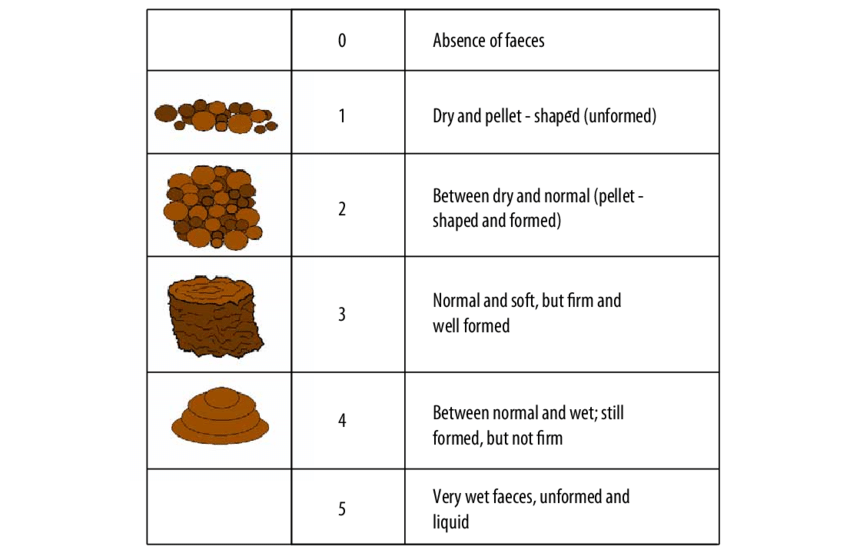


Faecal passage in transit: yes / no Faecal consistency (score):

Amount: nil / scant / ~one pile / multiple piles

**T1 faeces collected? (sample #)**

Urination: yes / no / uncertain

Meteorological information (source from BOM post travel):

|  | Departure | Way point #1 | Way point #2 | Way point #3 | Arrival |
| --- | --- | --- | --- | --- | --- |
| Location |  |  |  |  |  |
| Time |  |  |  |  |  |
| Temperature (^o^C) |  |  |  |  |  |
| Humidity |  |  |  |  |  |
| Rain / other  (detail) |  |  |  |  |  |

**Arrival abdominal ultrasound examination (T1)**

Date: _________________ Time: ________________________ Performed by: ___________________

Stomach US: not distended moderate distension marked distension

| Duodenal motility | Caecal motility | Jejunal motility | Ventral colon motility |
| --- | --- | --- | --- |
| *Visualized adjacent to R kidney* | *R lateral taenial band of caecum, ventral to costochondral junction* | *Image R or L inguinal region, or ventral abdomen* | *Image ventral abdomen, to right of midline* |
| Count number of circular contractions during 60s cineloop: | Grade movement:  0 = there is no movement  1 = there is infrequent (<2) mixing mvt evident  2 = there is 1 or 2 contractions (mvt away from probe / abdo wall) &/or frequent mixing contractions  3 = there are frequent contractions (>2) or continuous mixing movements | Grade visualization:  0 = no jejunal loops viz  + = 1 or 2 loops intermittently visible  ++ = 1 or 2 loops visible for most or all of video (60s)  +++ = multiple loops visualized  Grade movement:  0 = there is no movement  1 = mvt of ingesta <50% of recording  2 = mvt of ingesta >50% of recording  3 = near continuous motility  Sediment (yes/no) | Grade movement:  0 = there is no movement  1 = mvt of ingesta <50% of recording  2 = mvt of ingesta >50% of recording  3 = near continuous motility |
| File saved as*  DUOD _ _ _ _ _ | CAEC _ _ _ _ _ | JEJM _ _ _ _ _ | COLN _ _ _ _ _ |

**Post arrival cortisol swab sample:**

🞎 Time collected: _______________________

**Post-transportation veterinary examination (T1)** Time: _______________ Initials: ___________

HR __________ bpm MM colour and CRT: _____________________________

RR __________ bpm Peripheral perfusion, jugular refill: ___________________

Rectal Cardiac auscultation: _____________________________

Temperature _________ ^o^C Pulmonary auscultation: ___________________________

Abdominal auscultation (four quadrants):

0 absent

+ decreased

++ normal

+++ increased

Demeanour: ICS ___ / 2 min

LD RD

LV RV

Dull QAR BAR Excited

Other (specify)

Obvious injuries (describe) / other comments:

**Recovery ultrasound examination (T2)**

Date: _________________ Time: ________________________ Performed by: ___________________

Stomach US: not distended moderate distension marked distension

| Duodenal motility | Caecal motility | Jejunal motility | Ventral colon motility |
| --- | --- | --- | --- |
| *Visualized adjacent to R kidney* | *R lateral taenial band of caecum, ventral to costochondral junction* | *Image R or L inguinal region, or ventral abdomen* | *Image ventral abdomen, to right of midline* |
| Count number of circular contractions during 60s cineloop: | Grade movement:  0 = there is no movement  1 = there is infrequent (<2) mixing mvt evident  2 = there is 1 or 2 contractions (mvt away from probe / abdo wall) &/or frequent mixing contractions  3 = there are frequent contractions (>2) or continuous mixing movements | Grade visualization:  0 = no jejunal loops viz  + = 1 or 2 loops intermittently visible  ++ = 1 or 2 loops visible for most or all of video (60s)  +++ = multiple loops visualized  Grade movement:  0 = there is no movement  1 = mvt of ingesta <50% of recording  2 = mvt of ingesta >50% of recording  3 = near continuous motility  Sediment (yes/no) | Grade movement:  0 = there is no movement  1 = mvt of ingesta <50% of recording  2 = mvt of ingesta >50% of recording  3 = near continuous motility |
| File saved as*  DUOD _ _ _ _ _ | CAEC _ _ _ _ _ | JEJM _ _ _ _ _ | COLN _ _ _ _ _ |

**Recovery veterinary examination (T2)** Time: _______________ Initials: ___________

HR __________ bpm MM colour and CRT: _____________________________

RR __________ bpm Peripheral perfusion, jugular refill: ___________________

Rectal Cardiac auscultation: _____________________________

Temperature _________ ^o^C Pulmonary auscultation: ___________________________

Abdominal auscultation (four quadrants):

0 absent

+ decreased

++ normal

+++ increased

Demeanour: ICS ___ / 2 min

LD RD

LV RV

Dull QAR BAR Excited

Other (specify)

Obvious injuries (describe) / other comments:

**After transportation**

Location subsequent to transportation:

Arrival at destination:

Transport time from depot (approx.):

Appetite: nil (inappetant) reduced as expected increased

Provide details if abnormal:

Water consumption: not assessed reduced as expected increased

Demeanour: dull as expected agitated other (please describe)

Faecal output: not assessed reduced as expected increased

abnormal (describe):

Clinical abnormalities: colic diarrhoea febrile muscle soreness, tied up respiratory disease injury

(*circle any that apply, provide details below*)

Required veterinary attention: yes / no

(provide details)
