# Supplementary material for: Road transportation is associated with decreased intestinal motility in horses
Source: Front Vet Sci. 2025 Aug 18;12:1647236. doi: 10.3389/fvets.2025.1647236 (PMC12401009; doi:10.3389/fvets.2025.1647236)
Supplement: Supplementary file 2 [file Table_2.docx]

**Supplementary information 2 - Transport details**

Southbound trips departed from Williamstown depot in October and November 2022, with two four, three and four horses available for inclusion on each trip. Northbound trips departed from Tumbarumba in February and March 2023, with three, three and eight horses respectively. For the first northbound trip, an additional three horses were picked up from a second depot at Yass, approximately 2h north of Tumbarumba (six horses in total). A summary of trips included in the current study is provided in Table S2.1, with more detailed information included in Table 2.2, and the full data set uploaded as an Excel file.

Trucks departed between 03:30 and 06:00h, depending on direction of travel and transport depot. Horses arrived at the destination depot between 2:15pm (trip 8) and 5:05pm (trip 3).

Routes are illustrated in Figure S2.1, with an example of the travel data collected en route. Figure S2.2 shows horses undergoing sonographic assessment at Williamstown and Wangaratta depots.

*Table S2.1: Summary of trips included in current study (top) and overview of available data (bottom); a full data set is uploaded as an Excel file. (W’town = Williamstown depot; Tumba = Tumbarumba depot; Wang = Wangaratta depot).*

| Trip | Date | Number of horses | Depot | | Direction | Duration (h) | Distance (km) | Temp on arrival (^o^C) | Relative humidity on arrival (%) |
| --- | --- | --- | --- | --- | --- | --- | --- | --- | --- |
|  |  |  | Depart | Arrive |  |  |  |  |  |
| 2 | 11/10/22 | 2 | W’town | Wang | South | 10:15 | 830 | 20.3 | 49 |
| 3 | 15/11/22 | 4 | W’town | Wang | South | 11:54 | 840 | 19.9 | 57 |
| 4 | 23/11/22 | 3 | W’town | Wang | South | 11:25 | 800 | 18.4 | 47 |
| 5 | 29/11/23 | 4 | W’town | Wang | South | 10:55 | 1108 | 24.3 | 36 |
| 6 | 19/02/23 | 3 | Tumba | W’town | North | 13:15 | 760 | 31.6 | 45 |
|  |  | 3 | Yass |  |  | 9:47 | 456 |  |  |
| 7 | 26/02/23 | 3 | Tumba | W’town | North | 12:45 | 760 | 26.1 | 69 |
| 8 | 05/03/23 | 8 | Tumba | W’town | North | 10:45 | 703 | 37.2 | 24 |


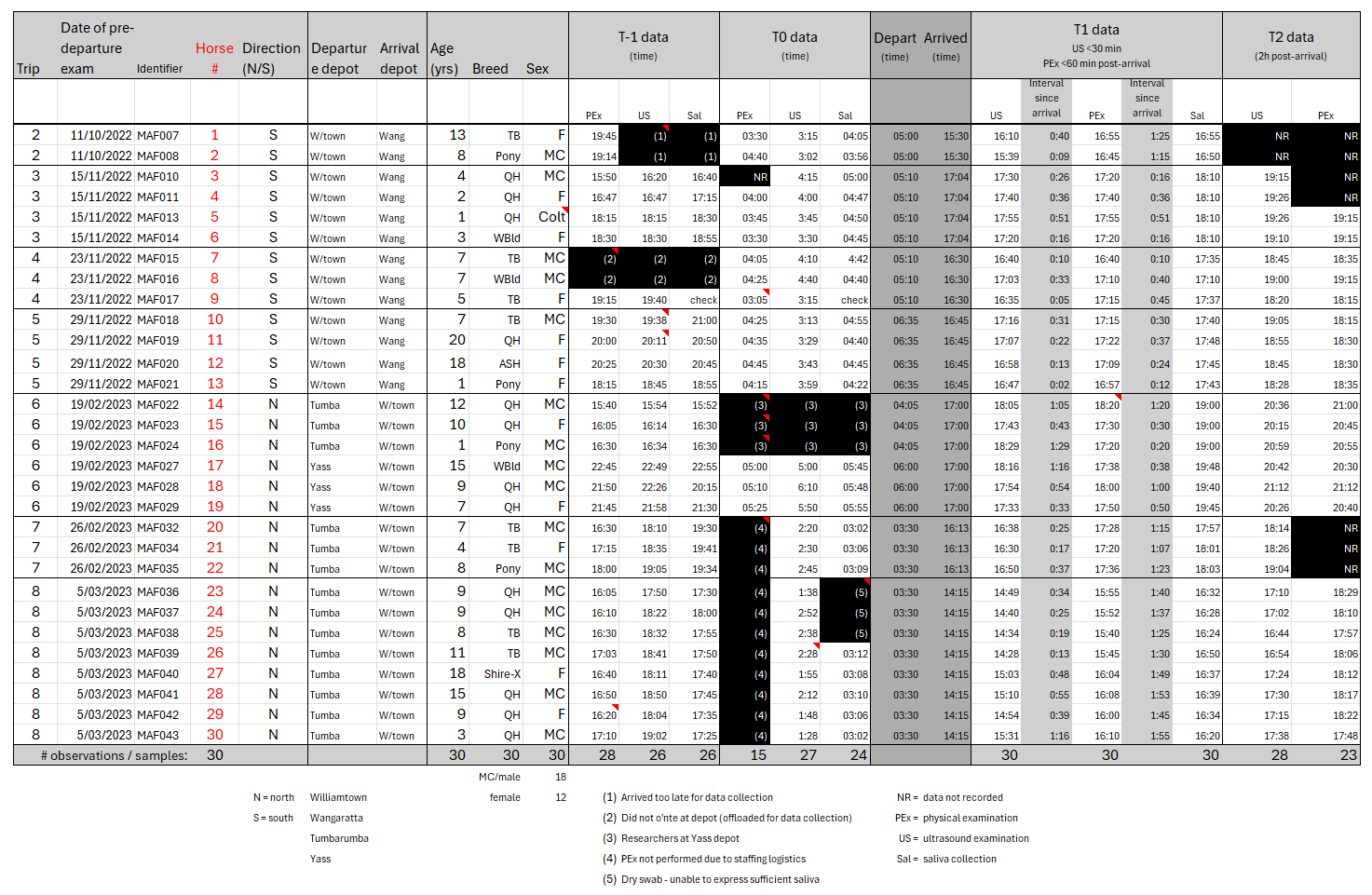
*Table S2.2: Overview of transport details and sample times for each journey. The full data set has been uploaded.*


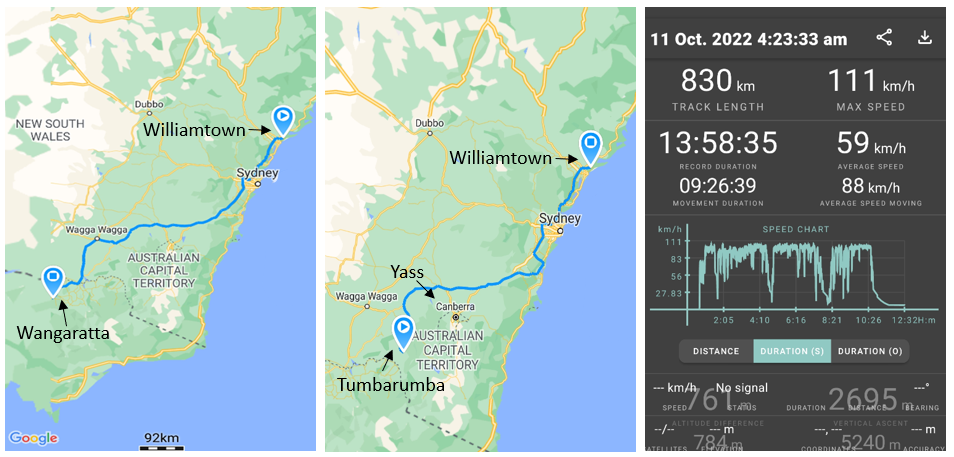


*Figure S2.1: Representative southbound (left) and northbound (middle) routes taken during study. The panel (right) shows data obtained from movement tracking of the transport vehicle.*


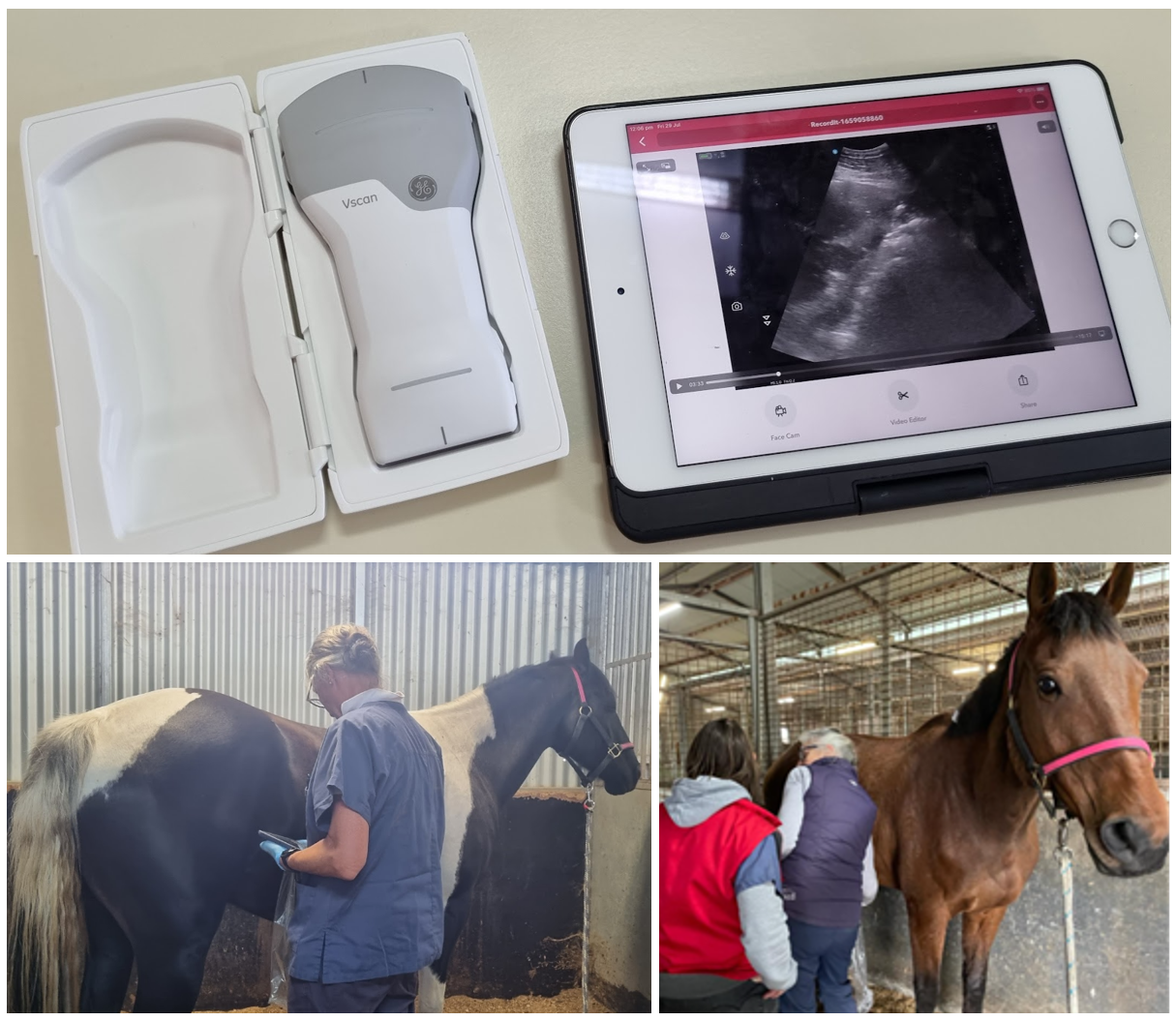


*Figure S2.2: VScan Air wireless ultrasound transducer (above) used in this project showing a representative image displayed on an iPad. Two study horses (below) standing without restraint for sonographic assessment of intestinal motility. Horses were very tolerant of this mode of assessment.*
