# Supplementary material for: Road transportation is associated with decreased intestinal motility in horses
Source: Front Vet Sci. 2025 Aug 18;12:1647236. doi: 10.3389/fvets.2025.1647236 (PMC12401009; doi:10.3389/fvets.2025.1647236)

**Veterinary examination findings**

*Pre-departure findings*

Of the study population, five horses were reported to travel frequently and fifteen travelled occasionally; past travel history was unknown for ten horses. Six horses were used for racing, two horses were performance horses or used for other competition but, for 22 horses, precise use was unknown or not specified. Three horses were sedentary or used infrequently, seven horses were exercising at medium or high intensity; exercise intensity was unknown for 20 horses.

Time at the depot was recorded for 28 horses (arrival time was not recorded for two horses that arrived at depot prior to researchers), and ranged from <1h to four days. Horses travelled with the transport company, owner or agent to arrive at the depot, after journeys lasting from 0.5 to 15h (mean 6.3h). Two horses on trip 4 (H23 and H24) were accommodated off site, at a location approximately 20 minutes from the depot, travelled with the driver to the depot on the morning of departure, and were not off-loaded at the depot.

**An over-view of pre- and post-travel observations is provided below to enable assessment of changes persisting across multiple observations times (Table 3.1), and a full data set has been uploaded.**

Physical examination at T-1 (evening prior to travel) was possible for the 28 horses accommodated at the depot, and was completed between 3:40pm and 10:45pm. Seventeen horses had normal demeanour (bright, alert, responsive), seven were considered quiet, and four appeared agitated. Minor abnormalities were noted on examination of 21 horses (supp info): eight had minor changes on auscultation of the respiratory tract (typically mildly increased adventitious sounds on tracheal auscultation); five were considered ‘tucked up’, an observation that reflected splinting of the abdominal musculature; four were considered to have slightly prolonged capillary refill or tacky mucous membranes; two had minor musculoskeletal abnormalities (long standing arthritic changes to one or more joints); two appeared foot sore (Obel grade 1) with mildly increased digital; two had faecal staining of their perineum and hindlimbs; and four had minor skin blemishes. Two horses demonstrated stereotypical behaviour (weaving, cribbing and/or windsucking). There were no abnormalities noted on cardiac auscultation for any horse. Jugular refill and peripheral perfusion were considered normal in all horses, and no horse was coughing. Abdominal auscultation was performed between 15 minutes and 6h after feeding, and no abnormalities were appreciated. No horse had decreased borborygmi in greater than two quadrants. Composite abdominal auscultation score was not related to time since feeding (r=-0.050, P=0.801) or time at depot (r=-0.77, P=0.697), but was weakly correlated to travel time to depot (horses that travelled further tended to have higher auscultation scores, r^2^ = 0.15, P=0.035). Heart rate (mean 38 bpm, range 28 – 52 bpm), respiratory rate (median 20 bpm, range 9 – 52 bpm) and rectal temperature (mean 37.7^o^C, range 36.9 – 38.9^o^C) were within reference ranges or elevated slightly, consistent with physiological responses to unfamiliar personnel and environment.

Demeanour at T0 (morning of travel) was assessed for 27 horses between 2am and 6am (the three horses loaded at Tumbarumba for trip 6 could not be assessed as researchers had travelled to Yass after completing T-1 data collection, in order to evaluate the remaining three horses in this consignment). Nineteen horses had normal demeanour (bright, alert, responsive), six were considered quiet, and two appeared agitated. Physical examination at T0 was completed for 16 horses, including the two horses (H23 and H24) not accommodated overnight at the depot. Veterinary assessment could not be standardised relative to feeding time. Heart rate (mean 41 bpm, range 32 – 58 bpm) was similar to values obtained at T-1, but RR (median 12 bpm, range 8 – 32 bpm) and rectal temperature (mean 38.0^o^C, range 36.6 – 38.3 ^o^C) were slightly lower than values obtained at T-1. Ten horses ate all feed offered overnight, eleven ate approximately half of the feed offered, and four horses ate little overnight; eleven horses drank more than 15L water overnight, thirteen horses drank approximately 5 to 15L, and two (H26, H25) drank <5L (observations were not recorded for three horses). Six horses ate all feed offered prior to departure, seven ate approximately half of what was offered, and twelve horses ate little feed prior to departure. All horses passed faeces overnight (one to five piles or dispersed throughout box), with faecal scores ranging from 1 to 5.

*Observations on arrival (T1)*

Seventeen horses had normal demeanour (bright, alert, responsive), eleven were considered quiet, one appeared dull, and one appeared agitated. Minor abnormalities were noted on examination of 12 horses (Table S3.1): one horse was coughing, four had minor changes on pulmonary auscultation of the respiratory tract, six were ‘tucked up’, six were considered to have slightly prolonged capillary refill or tacky mucous membranes, two had faecal staining of their perineum and hindlimbs, and two had scuffing or rubbing of their tails. One horse was pawing on stabling, and one had serous ocular and bilateral mucoid ocular discharge. There were no abnormalities noted on cardiac auscultation for any horse. Jugular refill and peripheral perfusion were considered normal in all horses. Heart rate (mean 42 bpm, range 32 – 60 bpm), respiratory rate (mean 24 bpm, range 12 – 60 bpm) and rectal temperature (mean 37.7^o^C, range 36.5 – 38.5^o^C) were within reference ranges or elevated slightly (Figure S3.1). Gastrointestinal borborygmi included gas sounds in four horses, and auscultation findings were significantly reduced in all four quadrants relative to pre-transport findings (Figure S3.2).

*Observations at T2*

Veterinary assessment or partial assessment was recorded for 23 horses at T2. Abnormal findings evident at T1 were largely resolved or unchanged (ie. none had progressed). Heart rate (mean 39 bpm, range 2 4– 50 bpm), respiratory rate (mean 21 bpm, range 10 – 38 bpm) and rectal temperature (mean 37.8^o^C, range 36.8 – 38.7^o^C) had largely returned to resting values (Figure S3.1). All but two horses had normal mucous membranes and abnormalities were not appreciated on thoracic auscultation of any horse. Abdominal auscultation grades remained decreased at T2 in the right ventral quadrant and based on composite score (Figure S3.2), and one horse still had gas on auscultation. Five of eight horses on trip 8 had abnormal findings at T2 including dermal lumps evident on his shoulder, inappetence, rolling, sweating and a tucked up appearance. The majority (22 of 28 horses) were observed to eat readily on arrival (six were initially hesitant), and the majority (22 of 28) drank readily on arrival (three drank a little, and three were not observed to drink); observations were not recorded for two horses. By the time researchers left (2 to 3 hours after arrival), all horses had eaten at least half of the feed offered, and all but two had drunk 5 to 10 litres of water. Ten (of 25) horses had passed at least one pile of faeces within 2 to 3h of arrival (not recorded for five), and faecal scores (median 4) were similar to pre-transport observations. No adverse health outcomes were recorded by owners or reported to the transport company associated with horse movements included in the current study.

*Table S3.1: Matrix of abnormal findings. Table constructed to facilitate recognition of abnormalities present at multiple times. Only abnormal findings have been recorded (blank cells indicate no abnormality detected); shaded cells indicate that assessment could not be completed at that time.*

*Figure S3.1: Rectal temperature, heart (HR) and respiratory (RR) rates determined prior to and following transportation. Data are shown in box and whisker plots with mean (+), median (horizontal line), quartiles (box) and range (whiskers) indicated, with all data points included.* *Pre-transport results (T-1 and T0) were combined using the higher value to give a single value for comparison with post-transport results; data from both time points are presented, but not included in comparisons. HR data was normally distributed and analysed by mixed effects model, with post-hoc testing using Tukey’s method and significant pairwise effects shown. RR and temperature data were not normally distributed and have been analysed using Kruskal Wallis test. A significant difference is evident between T-1 and T0 results for RR (P=0.020, determined by Wilcoxon test).*


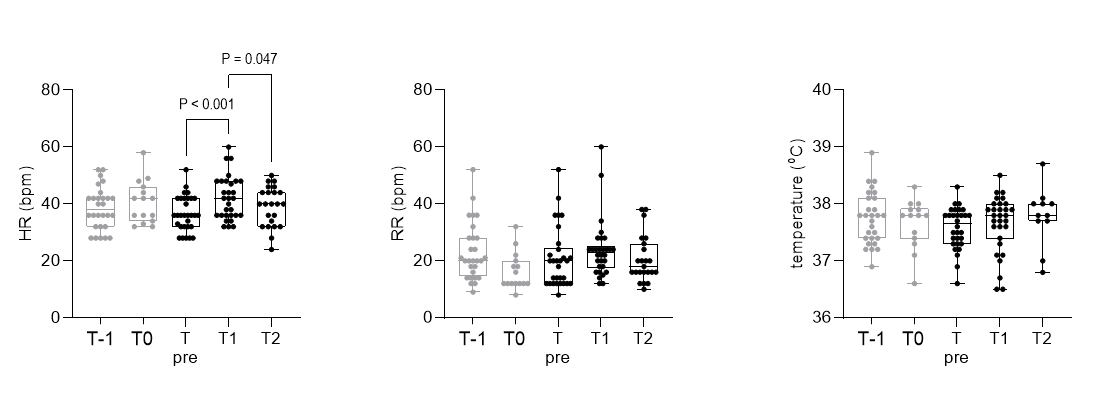


*Figure S3.2: Auscultation findings from the four abdominal quadrants prior to and following transportation. Borborygmi were graded in each quadrant (LD = left dorsal, RD = right dorsal, LV = left ventral, RV = right ventral): 0 = no borborygmi auscultated, 1 = decreased borborygmi, 2 = normal frequency and intensity of borborygmi, 3 = increased borborygmi. In addition, the number of ileocecal sounds (ICS) in 2 minutes were counted in the right paralumbar fossa. As graded data were ordinal variables and ICS were nonparametric, all data were analysed by Friedman test with post-hoc comparisons determined using Dunn’s method. Ordinal data are shown in violin plots with mean (red line) and quartiles (dotted line) shown; all continuous data are shown in box and whisker plots with mean (+), median (horizontal line), quartiles (box) and range (whiskers) indicated, and all data points included. Pre-transport results (T-1 and T0) were combined using the higher value to give a single value for comparison with post-transport results; data from both time points are presented, but not included in comparisons. Significant pairwise comparisons are shown.*


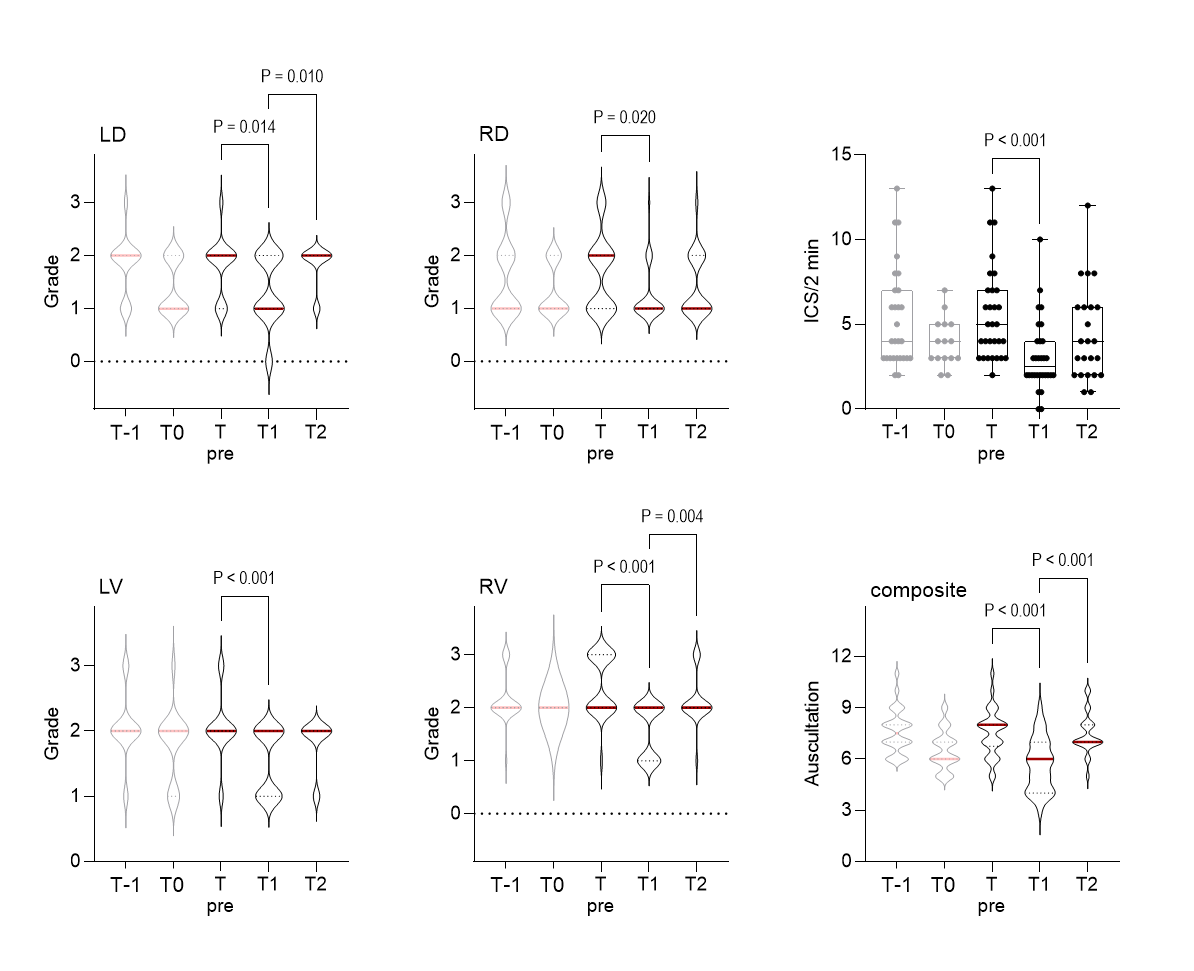

Supplement: Supplementary file 3 [file Table_3.docx]
