# Supplementary material for: Road transportation is associated with decreased intestinal motility in horses
Source: Front Vet Sci. 2025 Aug 18;12:1647236. doi: 10.3389/fvets.2025.1647236 (PMC12401009; doi:10.3389/fvets.2025.1647236)

**Pre-transport sonographic findings**

Ultrasonographic (US) assessment was possible for 26 horses at T-1 (two horses arrived at the depot too late to complete sonographic assessment, and two horses did not overnight at the depot, as noted above), and for 27 horses at T0 (three horses were loaded from the Tumbarumba depot when researchers had travelled to the Yass depot). Examinations were performed between 3:40 and 10:45pm (T-1), depending on arrival of horses into the departure depot, and prior to transportation (T0) between 1:28 and 6:10am, depending on scheduled departure time and researcher availability. Horses had been fed between 25 minutes and 6:55 hours prior to T-1 assessment. Six horses had not been fed prior to T0 examination, and 21 horses were examined between 0 and 2:28h after being fed. Where paired evaluations were completed, there was no difference between results obtained at T-1 in comparison with results obtained at T0 for DUOD (P=0.913, n=23), CAEC (P=0.965, n=23), JEJM (P=0.518, n=22), or when all motility grades were summed to give a composite score (P=0.051 n=22). Motility at T-1 was greater than that at T0 in COLN window (median difference 0.5, 98.3%CI 1.0 to 0, P=0.022). The time interval between feeding and sonographic assessment had no effect on the number of duodenal contractions (P=0.697) or DUOD grade (P=0.748), CAEC (P=0.973), COLN (P=0.900), JEJM (P=0.808), or on the composite motility grade (P=0.977).

Ultrasonographic assessment prior to transportation demonstrated a mid-gestation foetus in one horse (H30, Figure S4.1), a fact that was unknown to her owner or the transport company. One horse (H22) had an apparent increase in anechoic abdominal fluid and sonographic findings consistent with fibrin associated with abdominal structures. As this horse was otherwise normal on veterinary examination, and had no clinical history consistent with peritonitis, she was retained in the study. Haematology, performed at the request of her owner, demonstrated no abnormalities and she completed her planned journey without incident. One horse (H11) had been held in the depot for two weeks, with a vague history of ill-health. Veterinary examination details were not available for this horse and he was presented for travel at owner request. Veterinary and sonographic assessments demonstrated no health problems prior or subsequent to transportation in this horse.

Figure S4.1: Still image obtained from the COLN window at T-1 in H30 demonstrating a mid-late gestation foetus (note shadowing due to foal ribs).


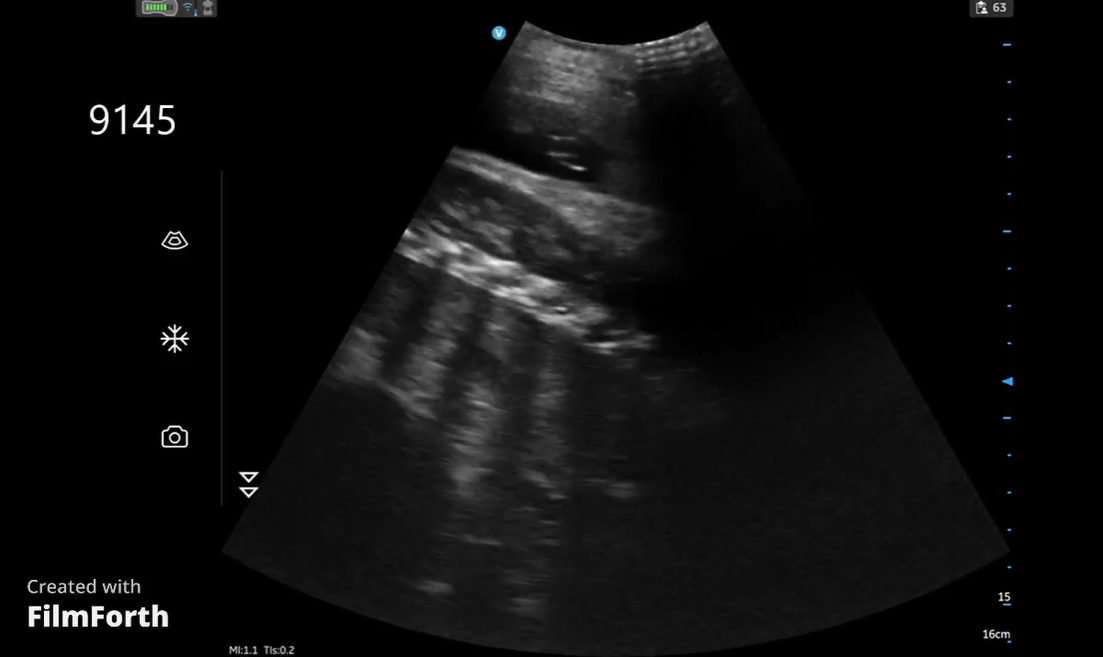


Figure S4.2: Still image from the COLN window of H22 demonstrating an apparent increased anechoic peritoneal fluid (arrow). Veterinary examination and haematology (performed at owner request) were not abnormal, and the horse completed her journey without adverse effect.


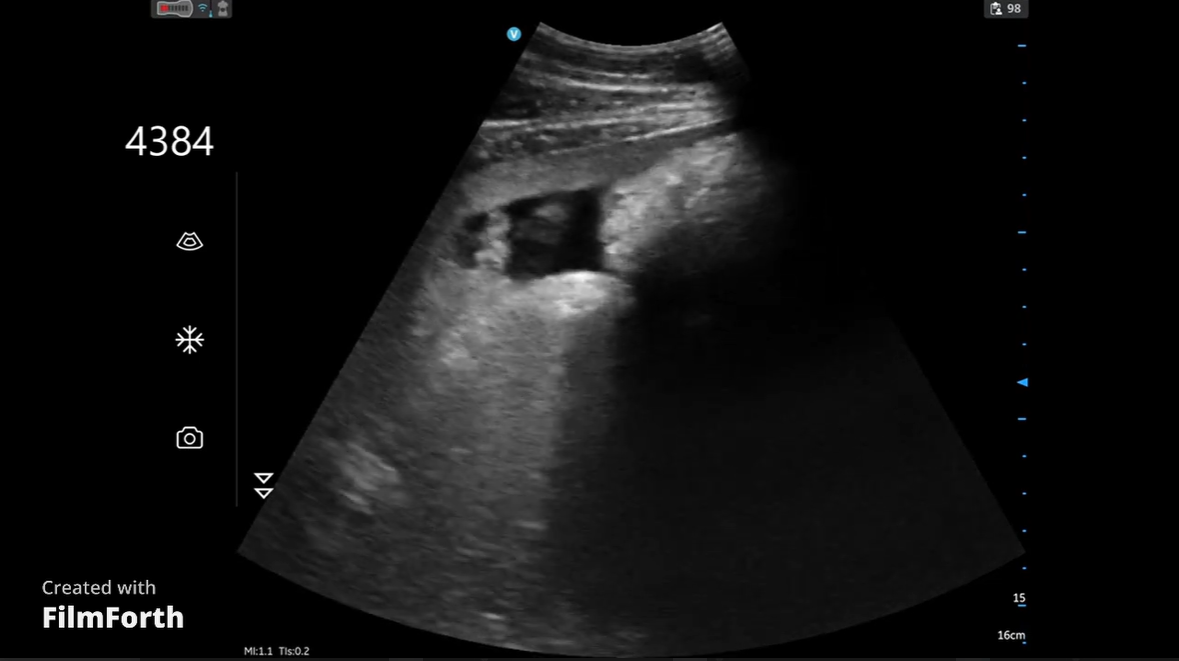

Supplement: Supplementary file 4 [file Table_4.docx]
