# Supplementary material for: Road transportation is associated with decreased intestinal motility in horses
Source: Front Vet Sci. 2025 Aug 18;12:1647236. doi: 10.3389/fvets.2025.1647236 (PMC12401009; doi:10.3389/fvets.2025.1647236)
Supplement: Supplementary file 5 [file Table_5.docx]

Table S1: Overview of factors included as explanatory and outcome variables. Comparisons between variables are indicated by a tick (🗸) or, for data collected on more than one occasion, the sampling time(s) (T-1, T0, T1, T2) evaluated is(are) specified.
