# Supplementary material for: Road transportation is associated with decreased intestinal motility in horses
Source: Front Vet Sci. 2025 Aug 18;12:1647236. doi: 10.3389/fvets.2025.1647236 (PMC12401009; doi:10.3389/fvets.2025.1647236)
Supplement: Supplementary file 6 [file Table_6.docx]

Table S2: Summary of trips included in current study (top) and overview of available data (bottom); a full data set is uploaded as an Excel file.

| Trip | Date | Number of horses | Depot | | Direction | Duration (h) | Distance (km) | Temp on arrival (^o^C) | Relative humidity on arrival (%) |
| --- | --- | --- | --- | --- | --- | --- | --- | --- | --- |
|  |  |  | Depart | Arrive |  |  |  |  |  |
| 2 | 11/10/22 | 2 | W’town | Wang | South | 10:15 | 830 | 20.3 | 49 |
| 3 | 15/11/22 | 4 | W’town | Wang | South | 11:54 | 840 | 19.9 | 57 |
| 4 | 23/11/22 | 3 | W’town | Wang | South | 11:25 | 800 | 18.4 | 47 |
| 5 | 29/11/23 | 4 | W’town | Wang | South | 10:55 | 1108 | 24.3 | 36 |
| 6 | 19/02/23 | 3 | Tumba | W’town | North | 13:15 | 760 | 31.6 | 45 |
|  |  | 3 | Yass |  |  | 9:47 | 456 |  |  |
| 7 | 26/02/23 | 3 | Tumba | W’town | North | 12:45 | 760 | 26.1 | 69 |
| 8 | 05/03/23 | 8 | Tumba | W’town | North | 10:45 | 703 | 37.2 | 24 |
